# The interdependence of mammary-specific super-enhancers and their native promoters facilitates gene activation during pregnancy

**DOI:** 10.1038/s12276-020-0425-x

**Published:** 2020-04-22

**Authors:** Xianke Zeng, Hye Kyung Lee, Chaochen Wang, Precious Achikeh, Chengyu Liu, Lothar Hennighausen

**Affiliations:** 1 Laboratory of Genetics and Physiology, National Institute of Diabetes, Digestive and Kidney Diseases, National Institutes of Health, Bethesda, MD 20892 USA; 2 Transgenic Core, National Heart, Lung, and Blood Institute, US National Institutes of Health, Bethesda, MD 20892 USA

**Keywords:** Gene regulation, Next-generation sequencing

## Abstract

Lineage-specific genetic programs rely on cell-restricted super-enhancers, which are platforms for high-density transcription factor occupation. It is not known whether super-enhancers synergize specifically with their native promoters or provide autonomous and independent regulatory platforms. Here, we investigated the ability of the mammary *Wap* super-enhancer to activate the promoter of the juxtaposed and ubiquitously expressed *Tbrg4* gene in the mouse mammary gland. The *Wap* super-enhancer was fused, alone or in combination with the *Wap* promoter, to the *Tbrg4* gene. While the super-enhancer increased the expression of the *Tbrg4* promoter five-fold, the combination of the super-enhancer and promoter resulted in 80-fold gene upregulation, demonstrating lineage-specific promoter–enhancer synergy. Employing ChIP-seq profiling to determine transcription factor binding and identify activating histone marks, we uncovered a chromatin platform that enables the high-level expression of the native promoter–enhancer but not the heterologous promoter. Taken together, our data reveal that lineage-specific enhancer–promoter synergy is critical for mammary gene regulation during pregnancy and lactation.

## Gene regulation: linking enhancers to specific promoters

Super-enhancers are regions of DNA that highly activate the expression of specific genes. Research led by H.K.L. and L.H. at the National Institutes of Health, USA, now demonstrate that a super-enhancer is much more effective when operating in synergy with its associated promoter, another gene-controlling element. They investigated super-enhancer and promoter synergy in mouse mammary gland cells. A mammary super-enhancer achieved a 5-fold activation of a gene with a common promoter, in contrast to an 80-fold activation with its own promoter together. The findings reveal that this synergy is critical for gene regulation in the mammary gland during pregnancy and lactation. They also help resolve the general issue of whether super-enhancers act independently or, as found here, can work together with a specific promoters in cell-specific genetic programs.

## Introduction

Super-enhancers (SEs) are key components in the control of lineage-specific transcription programs^1^. They feature platforms with a high density of transcription factors (TFs) and other regulatory components, such as RNA polymerase II (Pol II). However, it remains unclear why tissue-specific genes associated with structurally equivalent SEs are expressed at widely different levels, ranging by several orders of magnitude. SEs have been extensively investigated in the mammary gland, where they have been linked to genes induced to high levels of expression by lactation hormones during pregnancy^2^. The cytokine-induced signal transducer and activator of transcription (STAT) 5 TF is at the core of mammary SEs, which anchors additional TFs, including the glucocorticoid receptor and nuclear factor IB (NFIB)^2–4^. RNA-seq experiments have revealed that the expression of genes associated with mammary SEs ranges from less than one Fragments Per Kilobase of transcripts per Million mapped reads (FPKM) to more than 500,000 FPKM, and more than one-half of these expressed genes are not induced during pregnancy^2^. We speculated that this enormous diversity of gene expression is not only inherent to the structure and activity of the enhancers but is also caused by the interplay of specific and unique promoter–enhancers. Moreover, the impact of possible posttranscriptional regulation needs to be considered when assessing steady-state mRNA levels as surrogates for gene expression levels.

Although enhancer–promoter specificity was suggested by self-transcribing active regulatory region sequencing (STARR-seq) of *Drosophila*^5^ and the presence of promoter-specific transcriptional cofactors^6^, decisive genetic studies to validate promoter–enhancer synergy in vivo have not been conducted in mammals. As the mammary *Wap-*SE increases *Wap* gene expression by 1000-fold during pregnancy, we have investigated its ability, by itself and with its associated promoter, to induce the juxtaposed *Tbrg4* gene that is expressed in a wide range of cell types. Here, we employed CRISPR-Cas9 genome engineering in mice and placed the *Tbrg4* gene under the control of *Wap* regulatory elements. Our study provides genetic evidence that selective promoter–enhancer synergy is critical for the exceptional mammary-specific gene upregulation during pregnancy and lactation. Since the abundance of mammary-specific *Wap* mRNA during lactation cannot be attributed exclusively to transcriptional regulation, we also explored the possibility that the highly conserved 3′ untranslated region (UTR) plays a role. Finally, we provide evidence that the cAMP response element (CRE)-binding protein (CREB) TF is an integral part of non-mammary promoters and modulates the *Tbrg4* gene in vivo.

## Materials and methods

### Generation of mutant mice by CRISPR-Cas9

Six- to eight-week-old C57BL/6 female mice were purchased from Charles River. CRISPR/Cas9-targeted mice were generated by the Transgenic Core of the National Heart Lung and Blood Institute (NHLBI). All animals were housed and handled according to the guidelines of the Animal Care and Use Committee (ACUC) of the NIH (https://oacu.oir.nih.gov), and all animal experiments were approved by the ACUC of National Institute of Diabetes and Digestive and Kidney Diseases (NIDDK, MD) and performed under the NIDDK animal protocol K089-LGP-17.

CRISPR small-guide RNA (sgRNA) constructs were designed based on their proximity to the mutation sites and their off-target scores (calculated by the online tool at http://crispr.mit.edu/). Sequences of the specific sgRNAs used are listed in Supplementary Table 1. Each sgRNA was cloned into a pDR274 vector (Addgene #42250) separately, and injectable RNAs were transcribed in vitro using a MEGAshortscript T7 kit (Life Technologies). Cas9 mRNA was transcribed in vitro from plasmid MLM3613 (Addgene #42251) using the mMESSAGE mMACHINE T7 kit (Life Technologies). Zygote preparation and microinjections were performed as previously described. Superovulating C57BL/6 female mice were mated with C57BL/6 males, and the fertilized eggs were collected from oviducts. Then, 100 ng/μL of Cas9 mRNA and 50 ng/μL of each sgRNA in nuclease-free microinjection buffer (10 mM Tris [pH 7.5], 0.1 mM EDTA) were microinjected into the cytoplasm of the fertilized eggs. The injected zygotes were cultured overnight in M16 medium at 37 °C in 5% CO_2_. The next morning, the embryos that had reached the two-cell stage were implanted into oviducts of pseudopregnant recipients. All the mice were genotyped after PCR amplification of genomic DNA isolated from the tip of the tail and Sanger sequencing. The PCR and sequencing primers used are listed in Supplementary Table 2, and the Sanger sequencing results for the mutations are shown in Supplementary Tables 3 and 4.

### Chromatin immunoprecipitation sequencing (ChIP-seq) and data analysis

Frozen-stored mammary tissues and liver tissues harvested on day 14 of pregnancy (p14), day 1 of lactation (L1), and day 10 of lactation (L10) were ground into powder with a mortar and pestle. The chromatin was fixed with 1% formaldehyde at room temperature for 10 min, and the fixation was quenched with glycine at a final concentration of 125 mM. The samples were processed as previously described. The following antibodies were used for ChIP-seq: anti-STAT5 (Santa Cruz, sc-835 and sc-271542), anti-phospho-CREB (Millipore, CS204400), anti-H3K27ac (Abcam, ab4729), anti-H3K4me3 (Millipore, 07-473), and anti-RNA Polymerase II (Abcam, ab5408). Libraries for next-generation sequencing (NGS) were prepared as previously described and sequenced with HiSeq 2500 (Illumina).

Quality filtering and alignment of the raw reads was performed using trimmomatic^7^ (version 0.36) and Bowtie^8^ (version 1.1.2), with the parameter m1 selected to retain only uniquely mapped reads, using the mm10 reference genome. Picard tools (Broad Institute. Picard, http://broadinstitute.github.io/picard/. 2016) were used to remove duplicates, and subsequently, Homer^9^ (version 4.8.2) software was applied to generate bedGraph files. The integrative Genomics Viewer^10^ (version 2.3.81) was used for visualization. Each ChIP-seq experiment was conducted with two replicates.

### Pol II binding coverage and motif analysis

The reads from Pol II ChIP-seq covering each gene were then counted and normalized to those of the library size and gene length, and they were used to rank the transcription levels of SE-associated genes. Then, a motif search was performed with the promoters of the top 50 most-transcribed genes with those of the 50 least-transcribed gene promoters as background in Homer. Pol II and H3K27ac signaling on the promoters associated with different motifs was also profiled in Homer^9^.

### Total RNA sequencing (Total RNA-seq) and data analysis

Total RNA from the L10 mammary glands of Wap-Tbrg4 heterozygous mice was isolated using a PureLink RNA Mini kit (Ambion). RNA quality was measured by a bioanalyzer (Bio-Rad). One microgram of total RNA from each sample was used to generate the sequencing library with a TruSeq Stranded Total RNA Library Prep kit (Illumina) according to the manufacturer’s protocol. The RNA-seq library was sequenced with a HiSeq 2500 system (Illumina).

RNA-seq data were mapped to the mm10 reference genome using the STAR aligner^11^. The UCSC known gene database was used as a gene coordinate reference. To count the RNA-seq reads on exons and introns, the exons from all the transcripts of each gene were collapsed. The intron coordinates for each gene were then calculated based on the collapsed exon coordinates. HTSeq was used to count the RNA-seq reads on the exons and introns, and FPKM values were generated using the DESeq2 package in R (http://www.R-project.org)^12^.

### RNA isolation and quantitative real-time PCR (qRT-PCR)

Mammary tissues were harvested at the p14, L1, and L10 time points and then processed by an electronic homogenizer. Then, RNA was extracted using a PureLink RNA Mini kit (Ambion) according to the manufacturer’s instructions. cDNA was synthesized from total RNA using Superscript II (Invitrogen). Quantitative real-time PCR (qRT-PCR) was performed using TaqMan probes (Tbrg4, Mm01220234_g1; *Wap*, Mm00839913_m1, Thermo Fisher Scientific; mouse *Gapdh*, 4352339E, Applied Biosystems) on a CFX384 Real-Time PCR detection system (Bio-Rad) according to the manufacturer’s instructions. Tbrg4_Wap-fused mRNA and Wap 3′UTR-inserted Tbrg4-3′UTR mRNA were measured with the following respective primers using SYBR Green system: forward, 5′-TTTCCCAGTAATCCCAGTGC-3′, and reverse, 5′-CAAGGAGAAACGCTGTCTAGG-3′, and forward, 5′-CTGCAGGCTTCCTGGTAGTAGAT-3′, and reverse, 5′-CTTTTCGCATCTTGTCCTTGAG-3′. The PCR conditions were 95 °C for 30 s, 95 °C for 15 s, and 60 °C for 30 s for 40 cycles. All reactions were completed in triplicate, and the data were normalized to those of the housekeeping gene *Gapdh*. Relative differences in PCR results were calculated using the comparative cycle threshold (*C*_*T*_) method.

### Statistical analyses

For comparison of the samples, the data were presented with the standard deviation for each group and were evaluated with a *t-*test and ANOVA multiple comparisons using PRISM GraphPad software. Significance was obtained by comparing the measures from the wild type or control group and each mutant group. Values of *P < 0.05, **P < 0.001, ***P < 0.0001, and ****P < 0.00001 were considered significant.

## Results

### Promoter preference of the *Wap* super-enhancer

**Fig. 1:**
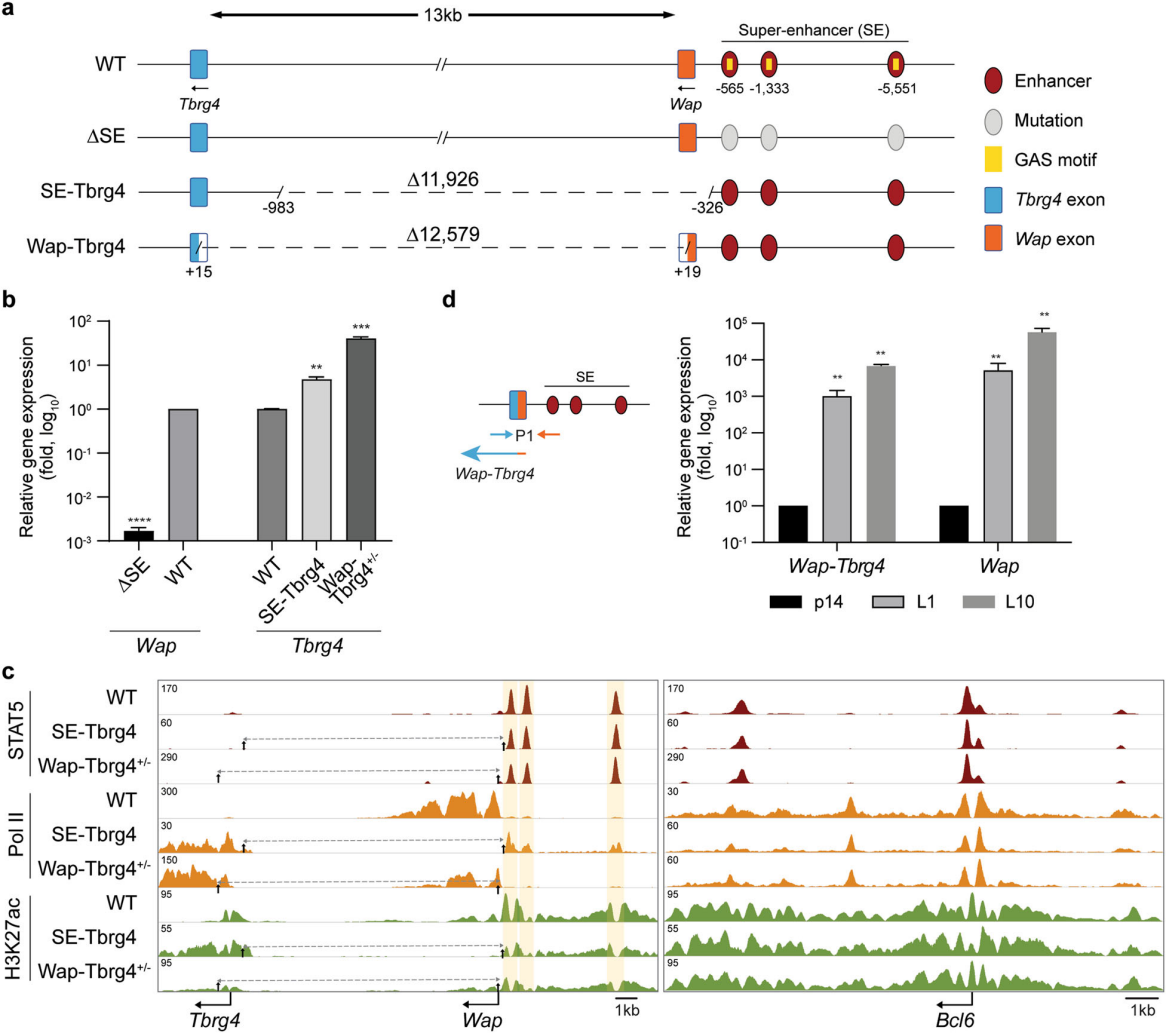
The functional establishment and activation of the *Wap* super-enhancer are dependent on the presence of its native promoter. **a** Schematic diagram of the region between the *Wap* super-enhancer and *Tbrg4* gene in the wild type (WT) and mutant mice. ∆SE mice were generated by deleting STAT5 binding sites in individual enhancers^2^. SE-Tbrg4 mutant mice were generated by deleting 11,860 bp spanning the sequence from −1048 upstream of the *Tbrg4* transcriptional start site (TSS) to +326 on the 5′ end of the *Wap* TSS (for detailed information on the sgRNAs and deletion break points, see Supplementary Tables 3 and 4). This deletion resulted in the translocation of the entire *Wap*-SE to the *Tbrg4* promoter. Wap-Tbrg4 mutant mice carried the deletion from the *Tbrg4* promoter to *Wap* promoter and the mutation fused the first exons of *Tbrg4* and the *Wap* genes. **b** *Wap* and *Tbrg4* expression levels in mammary tissues from the WT and mutant mice on L10 were measured by qRT-PCR and normalized to the *Gapdh* level. Results are shown as the means ± SEM of independent biological replicates (WT and mutants on L10, *n* = 3). ANOVAs were used to evaluate the significance of differences between the data obtained on L1 and L10 from the WT and mutants. **P < 0.001, ***P < 0.0001, ****P < 0.00001. **c** Genomic features of the *Wap-Tbrg4* locus in lactating mammary tissues (L10) of the WT and mutants. The magnified image of the *Tbrg4* TSS region is shown in Supplementary Fig. 1. The *Bcl6* locus was used as a control. The peak scales of each lane were determined based on that of the control locus and used to determine the biological significance of differences between the WT and mutant mice. **d** mRNA levels of the fused *Tbrg4* and *Wap* in heterozygous mammary glands from the Wap-Tbrg4 mouse line on p14 and L10 as measured by qRT-PCR. The respective locations of the primers are demonstrated.

The *Wap* gene, which provides as much as 5% of all transcripts in lactating mouse mammary tissue with expression induced more than 1000-fold during pregnancy, is controlled by prolactin and the TF STAT5 through a tripartite SE^2,3^ (Fig. 1a). In contrast, juxtaposed *Tbrg4* (*Fastkd4*) is ubiquitously expressed at low levels^13^. Here, we investigated whether the *Wap*-SE has an inherent preference for its associated promoter or has the capacity to effectively activate unrelated promoters. *Tbrg*4 and *Wap* are positioned in the same orientation and are separated by 13 kb. We used CRISPR-Cas9 to delete the sequences between the *Tbrg4* and *Wap* promoters in the mouse genome, thereby fusing *Wap* regulatory elements with the downstream *Tbrg4* gene (Fig. 1a). In the mouse line SE-Tbrg4, the tripartite *Wap*-SE was fused to the downstream *Tbrg4* promoter and positioned ~1000 bp from the TSS toward the 5′ end. In the line Wap-Tbrg4, the entire *Wap* regulatory region, including the SE, promoter and transcriptional start site (TSS), was translocated to the body of *Tbrg4*, thus generating a hybrid gene. An analysis of lactating mammary tissue from the SE-Tbrg4 mice revealed a five-fold increase in *Tbrg4* mRNA (Fig. 1b), demonstrating that lineage-specific *Wap*-SE has the capacity to activate a heterologous promoter. However, this activation was significantly lower than the 1000-fold upregulation of the endogenous *Wap* gene with the *Wap*-SE (Fig. 1b), suggesting the presence of additional key elements that communicate with this SE. To address this possibility, we studied the *Wap-Tbrg4* fusion gene, which was under the control of the *Wap* promoter–enhancer and thereby was expressed exclusively in the mammary tissue. Homozygous mutant fetuses died prior to embryonic day 8.5 (E8.5) (unpublished data), suggesting that *Tbrg4* expression in non-mammary cells is essential for embryonic development. To permit further analysis of the mutant allele, we generated compound heterozygous mice carrying one copy of the intact *Tbrg4* gene with a silent *Wap* gene and one copy of the *Wap-Tbrg4* fusion gene (Fig. 1a) that is expressed exclusively in mammary tissue. The chimeric *Wap-Tbrg4* mRNA levels from the mutant allele increased approximately 40-fold (Fig. 1b), which would be increased to 80-fold for two mutant alleles. These results demonstrate that the *Wap*-SE uniquely synergizes with the *Wap* promoter to execute maximum gene expression.

Next, we investigated the extent to which the *Wap*-SE was established in the absence and presence of its native promoter. For this, we determined TF-binding and histone modifications at the chimeric genes in lactating mammary tissue (Fig. 1c). In the *SE-Tbrg4* allele, all three *Wap* enhancers were occupied by STAT5, albeit to a lesser extent than they were in WT *Wap* alleles, suggesting that the SE structure can be established in the absence of the mammary-specific *Wap* promoter. While Pol II and H3K27ac coverage was very low in the WT *Tbrg4* gene (Supplementary Fig. 1), it was elevated on the *Tbrg4* gene under the control of *Wap*-SE (Fig. 1c). This finding provides further evidence that *Wap*-SE activates a foreign promoters. Pol II coverage at the three constituent enhancers within the translocated SE was similar to that at the WT locus (Fig. 1c).

Our earlier studies demonstrated that the integrity of the tripartite *Wap*-SE is critical for *Wap* gene activation at the onset of lactation^2^. However, as lactation is established, the most proximal constituent enhancer (E1) is no longer required, and the two distal enhancers (E2 and E3) are sufficient for *Wap* gene expression^3^. To evaluate whether the two distal enhancers have the capacity to activate the *Tbrg4* promoter, we deleted ~18 kb of DNA separating the *Tbrg4* promoter from its upstream region and the central constituent enhancer (E2) from *Wap*-SE (Supplementary Fig. 2a). ChIP-seq experiments demonstrated that STAT5 binding was established at E2 and E3 with a 75% reduction in coverage compared with that at the WT locus (Supplementary Fig. 2b). Thus, binding intensity was not sufficient to activate *Tbrg4* expression (Supplementary Fig. 2c). In summary, these data demonstrate that the integrity of the tripartite *Wap*-SE is essential for its activity and that the constituent enhancers E2 and E3 are functionally inactive despite their structural presence.

For the ChIP-seq analyses of mutant mammary tissue carrying one *Wap-Tbrg4* allele and one silent *Wap* allele, which does not have any enhancer marks (Fig. 1c), only the mutant allele was detected. The STAT5 binding and H3K27ac on the translocated *Wap*-SE and promoter were similar to the signals observed at the WT *Wap* alleles (Fig. 1c). Increased Pol II coverage coincided with increased expression. These findings underscore that the combined presence of the *Wap*-SE and its native promoter provides an ideal environment for the establishment of a functional regulatory complex and possibly promotes synergy between different regulatory elements.

A key feature of many mammary-specific genes is their exceptional upregulation during pregnancy, which further extends into the lactation period and can reach levels of several thousand-fold^3,14^. Given the landscape of STAT5 binding, H3K27ac marks and Pol II coverage on the *Wap-Tbrg4* mutant allele carrying the *Wap* promoter and SE, we surmised that the regulatory module requires key elements for the high *Wap* gene expression during pregnancy. We explored this possibility in mice that carry the *Wap-Tbrg4* mutant allele (Fig. 1d). *Wap-Tbrg4* chimeric mRNA levels increased ~1000-fold between day 14 of pregnancy (p14) and day 1 of lactation (L1) and another 5-fold at L10. *Wap* mRNA levels from the WT allele increased ~5000-fold between p14 and L1 and another 10-fold at L10 (Fig. 1d). These findings underscore that the synergy between the *Wap* promoter and its enhancer is critical for obtaining the extraordinary expression levels of the *Wap* gene observed during pregnancy.

**Fig. 2:**
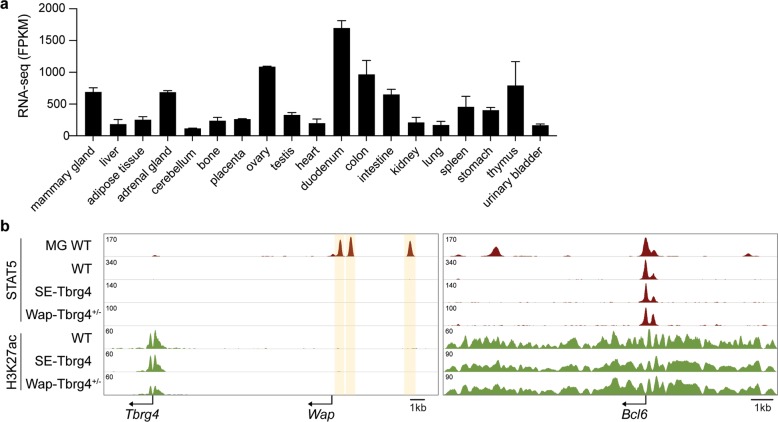
The translocated Wap super-enhancer is not established in liver tissue. **a** RNA-seq data from ENCODE demonstrate the presence of *Tbrg4* in multiple tissues of 8-week-old adult mice (https://screen.wenglab.org/search/?q=tbrg4&uuid=0&assembly=mm10). **b** Genomic features of the *Tbrg4*-*Wap* locus in the liver tissue from WT and mutants.

The common cytokine-inducible TF STAT5 is at the core of *Wap*-SE activity, which has been established only in mammary tissue. This finding begs the question of whether cell-specific open chromatin or additional mammary-specific factors are required for the establishment of *Wap*-SE. Because our mutant mouse lines carried *Wap*-SE adjacent to the constitutively active *Tbrg4* gene, we could address some of these questions. In contrast to *Wap*, the *Tbrg4* gene is expressed across many cell types, as evidenced by the RNA-seq data obtained from ENCODE (Fig. 2a). We analyzed the structure and function of translocated *Wap*-SE in the liver, a tissue that is highly enriched for activated STAT5. For this analysis, we performed ChIP-seq analyses of STAT5 binding and H3K27ac coverage (Fig. 2b). Neither STAT5 binding nor H3K27ac marks were detected at the *Wap*-SE that had been fused to *Tbrg4*. As a control, extensive STAT5 binding and H3K27ac were detected at regulatory regions of the *Bcl6* gene. These findings suggest that the presence of open chromatin is not sufficient for the establishment of the STAT5-dependent SE. It was hypothesized that additional mammary-enriched factors, such as NFIB or ELF5, are required for the establishment of a mammary SE.

### Posttranscriptional regulation of the *Wap* gene

**Fig. 3:**
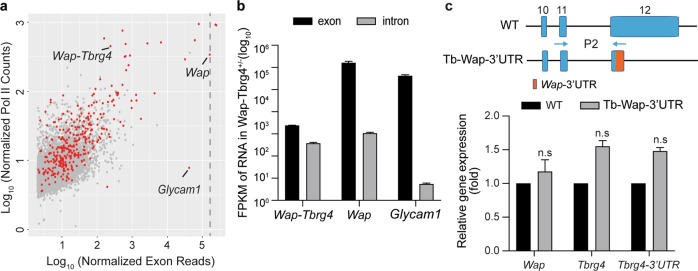
Gene induction during pregnancy and lactation is controlled by promoter and enhancer elements. **a** Scatter plot of normalized exon reads vs. Pol II ChIP-seq reads of expressed genes in the wt/Wap-Tbrg4 mammary tissue on L10. Red dots, SE-associated genes. **b** Expression levels of exons and introns in wt/Wap-Tbrg4 of mammary glands on L10 are shown, as determined by total RNA-seq. **c** *Wap*, *Tbrg4* and *Tbrg4-3*′*UTR* mRNA levels from WT and Tb-Wap 3′UTR mutant mice were measured by qRT-PCR on L1 and normalized to the *Gapdh* level. Results are shown as the means ± SEM of independent biological replicates (WT and mutants, *n* = 3). Multiple *t*-tests were used to evaluate the significance of differences between the WT and mutants. ns not significant.

Although the *Wap*-SE and its promoter are major transcription regulators, selective mRNA stabilization could contribute to these levels^15^. Notably, native *Wap* mRNA levels greatly exceed those of the *Wap-Tbrg4* fusion gene, where the *Tbrg4* coding region is under the control of the *Wap* promoter and SE. This divergence led us to investigate the contributions of transcriptional and posttranscriptional regulation to the expression of milk protein genes in lactating mammary tissue. We used Pol II loading as a surrogate measure for transcriptional activity. We quantified Pol II binding density over gene bodies and compared it with the mRNA levels for genes expressed in mammary tissue (Fig. 3a). Although Pol II loading on the *Wap-Tbrg4* allele and the native *Wap* allele were equivalent, the respective mRNA levels differed more than 100-fold (Fig. 3a). This finding suggested the possibility that selective mRNA stabilization might partially account for the high levels of some mammary-specific mRNAs.

To gain further insight into the regulation of mammary genes, we conducted total RNA-seq from mammary tissue on L10 and monitored nascent RNA levels by measuring intronic reads. In agreement with the Pol II loading, the nascent RNA levels of *Wap-Tbrg4* and *Wap* were similar (average FPKM of 37.5 vs. 106.6) (Fig. 3b and Supplementary Table 4), indicating comparable transcription of the two genes. Notably, steady-state mRNA levels of the mammary gene *Glycam1* were also extremely high in the L10 mammary tissue (average FPKM of 41,824). However, the levels of Pol II loading and *Glycam1* transcription were low (average nascent RNA FPKM 0.5) (Fig. 3a, b and Supplementary Table 5), suggesting that not all mammary genes are controlled by the same mechanisms.

In support of the posttranscriptional regulatory mechanism, experiments with transgenic mice suggested that the highly conserved 3′UTR of the rat *Wap* gene contributes to the high mRNA levels in mammary tissue^16^. We therefore investigated the potential role of the highly conserved mouse *Wap*-3′UTR^17^ in regulating *Tbrg4* mRNA levels. For this experiment, we replaced the *Tbrg4*-3′UTR with the entire *Wap*-3′UTR in the mouse genome (Fig. 3c) and measured mRNA levels in lactating mammary tissue. Levels of the *Tbrg4-Wap* chimeric mRNA were similar to those of the WT *Tbrg4* mRNA, suggesting that the *Wap*-3′UTR by itself did not impose measurable mRNA stabilization.

### CREB function at the Tbrg4 promoter

**Fig. 4:**
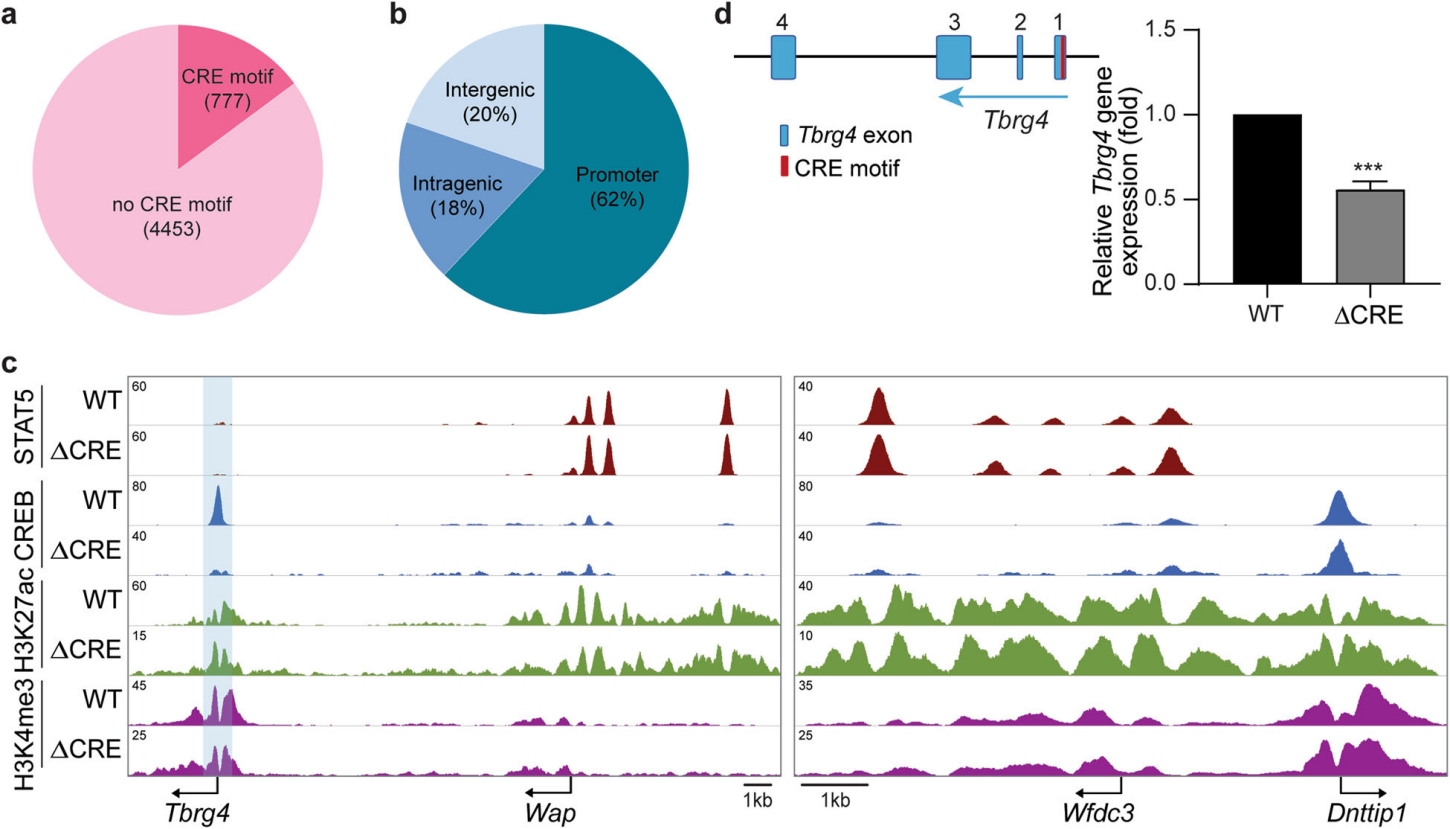
Role of the CREB transcription factor at the *Tbrg4* promoter. **a** ChIP-seq experiments determined a total of 5230 genomic sites binding CREB in lactating mammary tissue, 777 of which contain a CRE motif. **b** Percentage of binding sites based on their genomic locations. **c** ChIP-seq profile of the *Tbrg4*-*Wap* locus in WT and ΔCRE mutant mammary tissue on L1. The *Wfdc3-Dnttip1* locus served as a control. **d** *Tbrg4* mRNA levels in the mammary tissues of the WT and ΔCRE mutant mice on L1 were measured by qRT-PCR and normalized to *Gapdh* level. Results are shown as the means ± SEM of independent biological replicates (WT and ΔCRE, *n* = 5). *t*-tests were used to evaluate the significance of differences between the WT and mutants. ***P < 0.0001.

The *Tbrg4* and *Wap* promoters respond differently to the *Wap*-SE, suggesting that they are recognized by unique sets of TFs that can synergize with their respective enhancers. To verify this supposition, we conducted a genome-wide search for TF-binding motifs. The motif for the CREB transcription was greatly enriched in genes not highly expressed in mammary tissue. To validate CREB binding in vivo, we conducted ChIP-seq experiments using lactating mouse mammary tissue. Out of the 5230 genuine binding regions, only 777 coincided with a CRE motif (Fig. 4a), suggesting that the vast majority of CREB binding occurs through other TFs or cofactors. The majority of binding sites were associated with promoter regions (Fig. 4b), suggesting a key role in promoter activation. Strong CREB binding to the consensus motif TGACGTCA was observed at the *Tbrg4* promoter (Fig. 4c), suggesting that this site might be critical for *Tbrg4* regulation and that it possibly shields the *Tbrg4* gene from *Wap*-SE. We tested this hypothesis and inactivated the CRE motif within the mouse genome (Fig. 4d). Deletion of this site resulted in the loss of CREB occupancy in lactating mammary tissue (Fig. 4c) and an approximate 45% reduction in *Tbrg4* mRNA levels (Fig. 4d). This finding demonstrates that CREB is not essential for *Tbrg4* gene expression but rather modulates its activity. ChIP-seq experiments further demonstrated that H3K27ac and H3K4me3 marks on the *Tbrg4* gene and the neighboring *Wap* gene were not altered in the absence of CREB binding (Fig. 4c), supporting a limited function of CREB in establishing promoter activity or regulating nearby enhancers.

## Discussion

Gene expression is largely controlled by two types of regulatory elements, promoters and enhancers. Although this pattern is true for some genes, the annotation and definition of regulatory elements is currently being expanded, as enhancers have been identified in promoters and enhancers can contain promoters^18,19^. Since enhancers can activate genes over long distances, assigning individual enhancers to their respective target genes remains a challenge. Classical assays, including recent studies using integrated reporters in cell lines, merely provide evidence that enhancers have the capacity to activate generic promoters, but they provide limited information on the genuine target promoters. Chromatin capture technologies have provided detailed snapshots of the areas proximal to enhancers with promoters^20–22^, but the functional significance of their placement has been rarely investigated in true in vivo settings within an intact animal.

The concept of enhancer–promoter interdependence has been studied and debated extensively^18,19^. However, there is limited in vivo evidence that enhancers preferentially activate the promoters of their true target genes and that they have limited activity on unrelated promoters. Our study provides evidence that a mammary SE preferentially regulates its own target promoter. Although translocated mammary SE specifically activated an unrelated ubiquitous promoter in mammary tissue, the native locus demonstrated clear enhancer–promoter interdependence with a 20-fold higher expression. Importantly, the native enhancer–promoter unit regulates an unrelated gene, suggesting that it contains most, if not all, the regulatory elements to respond to hormonal cues in the mammary gland during pregnancy and lactation. At this point, the mechanistic underpinning of enhancer-promoter interdependence is not fully understood. In vitro experiments point to the presence of specific cofactors recruited by different classes of promoter elements^6^. It remains to be determined whether mammary-specific promoters bind defined classes of cofactors. This study also extends our recent findings showing that mammary *Wap*-SE has limited capacity to activate the unrelated neighboring *Ramp3* promoter, both in the endogenous setting^13^ and upon genomic translocation^3^. The extent to which *Wap* promoter-SE sequences activate the unrelated *Tbrg4* gene is less than that of the activation observed at the endogenous *Wap* locus, suggesting the possible presence of additional regulatory elements within the *Wap* gene or in the intergenic region between *Wap* and *Tbrg4*. However, detailed ChIP-seq analyses for several binding TFs and Pol II and activating histone marks failed to provide evidence for additional transcriptional regulatory elements (Supplementary Fig. 3).

Although expressed in mammary tissue, the *Tbrg4* gene is not induced during pregnancy, suggesting that it is not under the control of *Wap*-SE^2^. At this point, it is not clear why *Tbrg4* is outside the sphere of influence of *Wap*-SE. SEs are frequently located within chromatin loops that are anchored by the CTCF protein, which might confine enhancer activity to genes within these loops^23–27^. In support of this idea, disruption of CTCF sites in mice has led to ectopic expression of genes outside the topologically associating domain (TAD) or sub-TAD^28,29^. In a previous study^13^, we identified two CTCF sites that coincide with a TAD boundary^30^ and separate the *Wap* gene from the *Tbrg4* gene. Deletion of the two CTCF sites did not result in altered expression of either *Tbrg4* or *Wap* in lactating mammary tissue^13^, demonstrating that CTCF at either site does not insulate the *Tbrg4* gene from *Wap*-SE. Since no additional potential insulating elements were identified between *Wap*-SE and *Tbrg4*, we speculate that distance might play a larger role than previously thought. Alternatively, *Wap*-SE might preferentially recognize the most proximal promoter.

Our findings that enhancer-promoter synergy determines transcription output provide an explanation for the distinct transcriptional levels of SE-associated genes. The expression of the more than 400 genes that are under the control of mammary SEs varies over several orders of magnitude, suggesting the presence of additional promoter-bound TFs that can greatly boost mammary SE activity. We identified CREB TF binding in non-mammary promoters but not in promoters selectively active in mammary tissue, suggesting that they might suppress the promoter response to mammary SEs. However, genetic ablation of CREB binding on a non-mammary promoter did not support this concept.

Differential mRNA half-lives were discovered decades ago^31,32^. Similar to milk mRNAs stabilized by hormones in mammary tissue, vitellogenin mRNA is stabilized by estrogen in the liver, while interferon and IL-2 are stabilized by CD3 and CD28 receptor involvement in T cells^33,34^. However, the mechanisms of hormone-mediated RNA stabilization are still unclear. Hormones may trigger specific RNA-binding proteins to protect mRNA from degradation or activate/inhibit specific small RNAs that regulate RNA decay^32,35^. Lineage-specific and gene-specific mRNA stabilization probably involves both of these processes and requires further investigation.

While our study focused on understanding the mechanistic aspects of the *Wap* gene, including its promoter and SE, it also shed light on the *Tbrg4* gene and its role in mammalian development. TBRG4, also known as FASTKD4, is transported to mitochondria and serves as a key posttranscriptional regulator of mitochondrial gene expression^36^. Notably, it appears to control the half-lives of mitochondrial RNAs^37,38^. In cell lines, CRISPR-mediated targeting of the *Fastkd4* locus resulted in an apparent absence of the corresponding protein and decreased levels of mitochondrial RNA, but no aberrant cell viability was reported^36^. *Tbrg4* is expressed across all cell types, and in our study, the *Tbrg4* allele was expressed only in the mouse mammary epithelium. While the heterozygous mice were viable and indistinguishable from the WT mice, homozygous mutant mice were not viable, with the fetuses dying prior to implantation, demonstrating that *Tbrg4* function is essential for early embryonic development. It is speculated that impaired mitochondrial physiology causes the early embryonic death. Our mouse study highlights differences in the requirements of this mitochondrial regulator in the fast-growing embryo compared with those in the immortalized cell lines that survive without TBRG4^39^.

## Supplementary information

Supplementary information

Supplementary Table 5

## Data Availability

All data were obtained from or uploaded to the Gene Expression Omnibus (GEO). ChIP-seq and RNA-seq data on wild type and *Wap* super-enhancer mutant tissue were obtained from the GSE74826, GSE115370 and GSE127139 data sets. The RNA-seq data for the mutant mice generated in this study were uploaded to GSE121438. The ChIP-seq data have been deposited to GSE145193.
