# Supplementary material for: The interdependence of mammary-specific super-enhancers and their native promoters facilitates gene activation during pregnancy
Source: Exp Mol Med. 2020 Apr 22;52(4):682–90. doi: 10.1038/s12276-020-0425-x (PMC7210877; doi:10.1038/s12276-020-0425-x)
Supplement: Supplementary file 1 — Supplementary information [file 12276_2020_425_MOESM1_ESM.pdf]

Supplementary information

Interdependence of a mammary-specific super-enhancer and its native promoter facilitates gene activation during pregnancy

Running title: Synergy of lineage-specific regulatory elements

Total number of words: 3300

This work was supported by the Intramural Research Programs (IRPs) of National Institute of Diabetes and Digestive and Kidney Diseases (NIDDK) and National Heart, Lung, and Blood Institute (NHLBI).

Xianke Zeng[1, #], Hye Kyung Lee[1, #, *], Chaochen Wang[1, #], Precious Achikeh[1], Chengyu Liu[2], and Lothar Hennighausen[1, *]

[1]Laboratory of Genetics and Physiology, National Institute of Diabetes, Digestive and Kidney Diseases, National Institutes of Health, Bethesda, MD 20892, USA

[2]Transgenic Core, National Heart, Lung, and Blood Institute, US National Institutes of Health, Bethesda, MD 20892, USA

* Correspondence to: H.K.L (hyekyung.lee@nih.gov) and L.H (lotharh@niddk.nih.gov)

[#] Co-first authors

Supplementary Figures

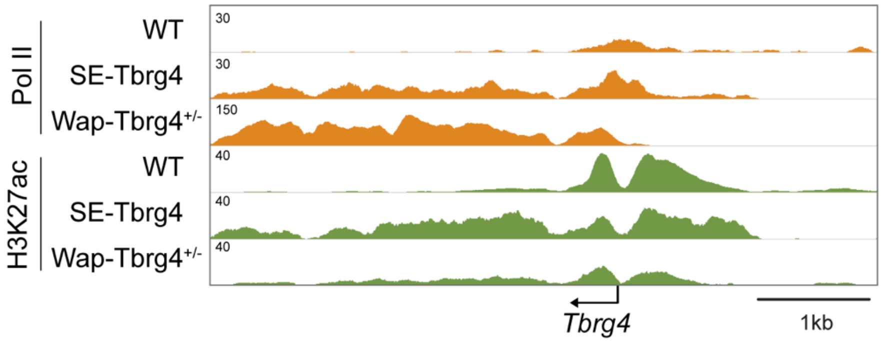

Supplementary Fig. 1 ChIP-seq data of the *Tbrg4* promoter and transcriptional start site (TSS) region in mammary tissue at day 10 of lactation (L10) from WT mice and mutants.

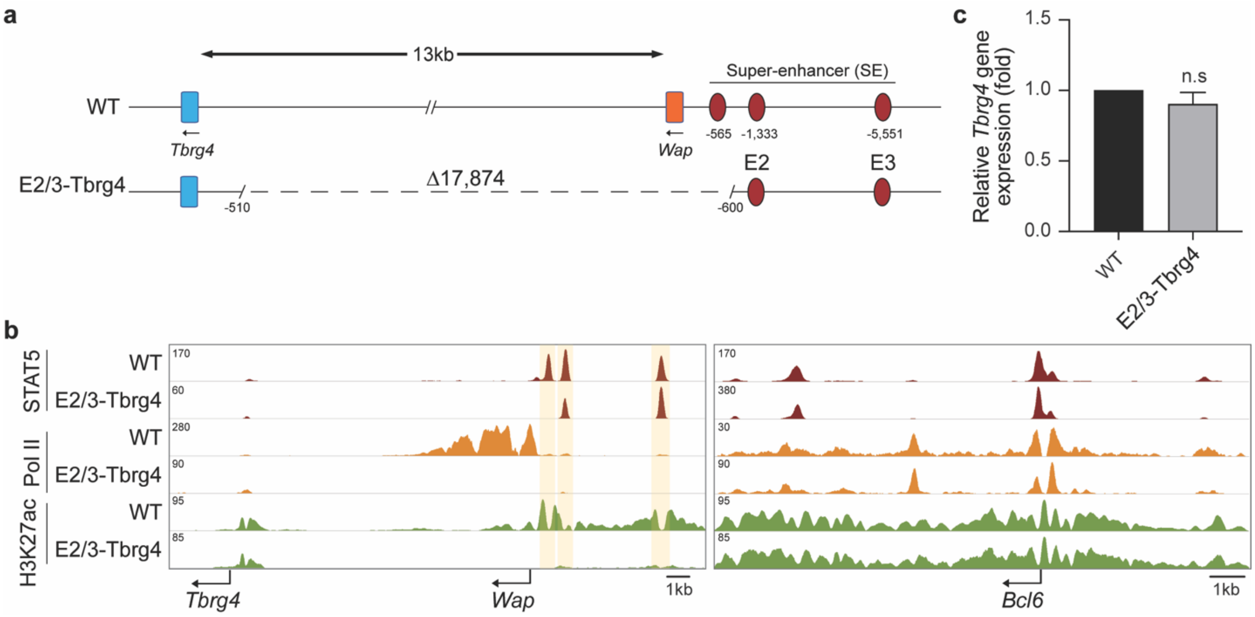

Supplementary Fig. 2 Capacity of *Wap* super-enhancer elements E2 and E3 to activate the *Tbrg4* gene. a Diagram of mutants in which *Wap* enhancers E2 and E3 were fused with the *Tbrg4* upstream region (E2/3-Tbrg4). By deleting a 17.874 kb stretch of DNA, the *Wap* enhancers E2 and E3 were translocated within 510bp of the *Tbrg4* transcription start site. The exact positions of the deletions are shown in Supplementary Table 3 and 4. **b** STAT5A, RNA Pol II and H3K27ac landscapes at the *Tbrg4*-*Wap* locus in WT and E2/3-Tbrg4 mutant mammary tissue at day 10 of lactation (L10). The *Bcl6* locus was used as a control. **c** *Tbrg4* mRNA levels from WT and E2/3-Tbrg4 mutants at dat 10 of lactation (L10) were measured by qRT–PCR and normalized to *Gapdh* levels. Results are shown as the means ± s.e.m. of independent biological replicates (WT and E2/3-Tbrg4 at L10, n = 4). A t-test was used to evaluate the statistical significance of differences between WT and mutant mice. n.s. not significant.

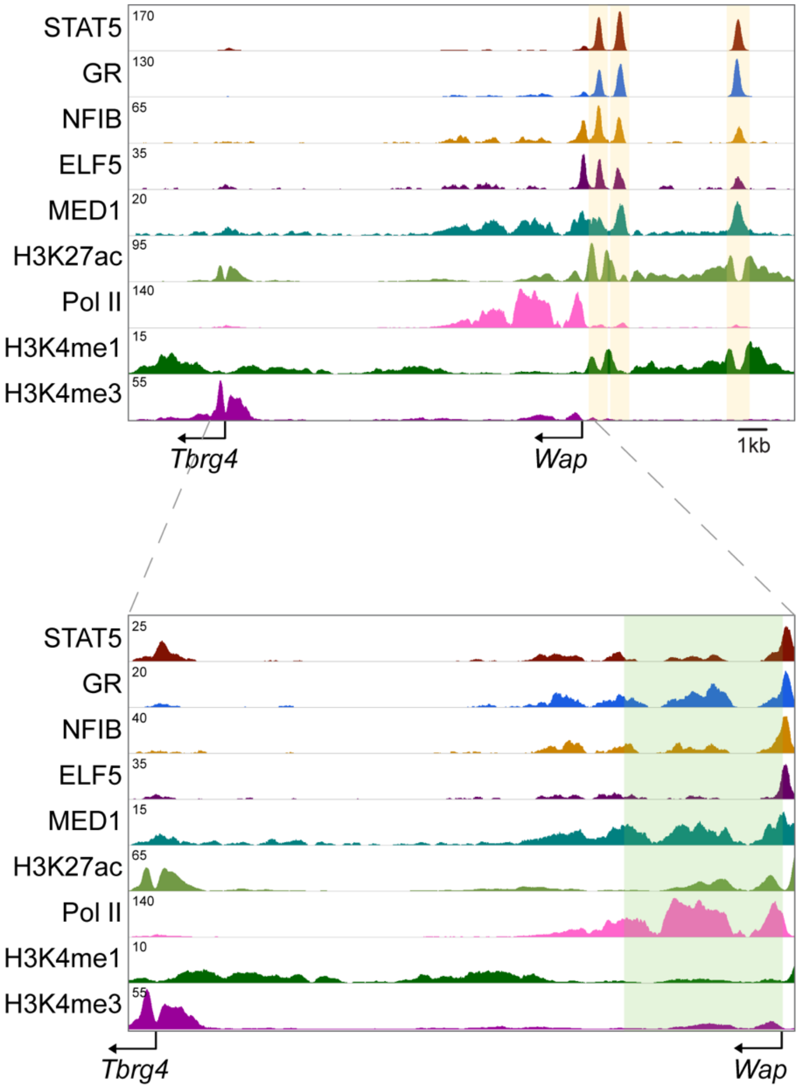

Supplementary Fig. 3 ChIP-seq profiles for the *Tbrg4*-*Wap* locus and regulatory elements in mammary tissue at day 10 of lactation (L10) from WT mice. The area shaded green covers the body of the *Wap* gene.

Supplementary Tables

Supplementary Table 1. Sequences of sgRNA for CRISPR/Cas9 targeted mice

Tbrg4_sgRNA	sgRNA sequences
SE-Tbrg4	5’-GCCAGCAGGATAACTTAGTG-3’<br>5’-CAAGTGTCACTTTGAGTGGT-3’
Wap-Tbrg4	5’-CAACGCATGGTACCGGTGTCAGG-3’<br>5’-GTGACGTCAGTGAGCGCGCCAGG-3’
E2/3-Tbrg4	5’-CAACGCATGGTACCGGTGTCAGG-3’<br>5’-CAGGGCCTCCACAGTAGTAC-3’
CRE	5’-TGACGTCACTGGCGCGCCGCCGG-3’<br>5’-GTGACGTCAGTGAGCGCGCCAGG-3
Tb-Wap 3’UTR	5’-AGAAAAGCCAGTGGCCCATGTGG-3’<br>5’-GCAGTCTGAACTGATGGGTAAGG-3’<br>Donor 5’-GAAAAGCAATGGCTGAGGAGCTAGCCAAATGACCT<br>GCCGGCCCTGGCATCCCTGCCTGTCGGGAGTGACCAGC<br>CCAAGTCTATACAGCAAGACCCTTCACTCCTGGATCCAG<br>AGAGAACATAATGCTTTCAATCTGCTGCTAATAAAAATCCA<br>TTTGGCTTTATG-3’

Supplementary Table 2. Genotyping primers for CRISPR/Cas9 mice

Mutant mouse line	PCR and Sequencing primers
SE-Tbrg4	Forward 5'-ACGCCAAGGGTCACTGCG-3'<br>Reverse 5'-GCAAGATGCAGAGAGAACAGAGC-3'
Wap-Tbrg4	Forward 5'-AAGCTAGGTGGCCCAGTGCTC-3'<br>Reverse 5'-GCAAGATGCAGAGAGAACAGAGC-3'
E2/3-Tbrg4	Forward 5'-ACGCCAAGGGTCACTGCG-3'<br>Reverse 5'-GCAAGATGCAGAGAGAACAGAGC-3'
CRE	Forward 5'-TCGGTTGCAAATTCGGTTCC-3'<br>Reverse 5'-GTAGACGGGAAGGAGAGGGT-3'
Tb-Wap 3'UTR	Forward 5'-GAACTACTGCAGAGCCACCC-3'<br>Reverse 5'-GGCGCTTTTCTGCCATTCAG-3'

Supplementary Table 3. Breakpoint of mutant mouse line in *Tbrg4* and *Wap* loci

Mutant mouse line	Wap breakpoint	Tbrg4 breakpoint	Deletion size
SE-Tbrg4	-326	-983	11,926
Wap-Tbrg4	+19	+15	12,579
E2/3-Tbrg4	-842	-511	17,874
Tb-Wap 3'UTR	-	+9475	1,080
CRE	-	-20 / +2	22

Supplementary Table 4. DNA sequences of mutant mice.

Wap-SE

GAS motif

TSS of *Wap* gene

TSS of *Tbrg4* gene

SE-Tbrg4 (Δ11,926)

TCAGGTTTTTAGGGAAAATTCCAGCACAGCTGACCCCATAGGACAAAAAGTGCAGTGTGTGTACTACCAA
AACCCTGGTGTCCTTTTCCCACCCTAGCCGAGCAGAGTTGATGGGGCAGGAAAGAGCCTACGCATACTGG
AAGCACACAGGCTCAAGACTGGCAGGCCAAAGAACCAGAACACCCCAGGGCATAAGAACCCCATGCCCCT
TGCCGCTGGGCCTGGTGAATTCAGAGTAATGTCTTCA**TTCCTAGAA**CCTTCTGGCTTCCCGACCTGGCCT
GCAGGCTCTGAGGAGATGTGTGCACCTCATGAACTCCTTGTTCAGCCAGGGCCTCTCTGTCCTCCCTACA
CTTCCCCACCACACAGGAACACATGTCCTCAACTGACCAGTGTCACCCTGGGCCTCAAGGCTAGGTTCCC
TTGAGTACTGGGACACAAGAACAGACTTCTGCTCTCCCCTCTGTCCACACAGAGTGCAGAGAGGAAGGGT
TCTGGTTCATGTCCCACAGTTGCCCCCTAAAACCGATGTGATATAGCCCTCACTGGCCTAGAGCTCACTA
CATAAACCAGGCTGGCCTGGAACCCACAGATCTGTCTGCCTTTGACTCCTACTGCTGGGATCAAAGGTGT
GAGCCACCATGCAACGCTCTGAAACTGATTCTTTAGAAGCTAAGAAAATGCTTCAAAATGGCAGTAGCCT
TGGGCTGGAGATGGGTTAGTGGTTAAGAGAACTGACTGCTCTTCCAGAGAGAGAGAGAGAGAGAGAGAGA
GAGAGAGAGAGAGAATGAATGAATGAATCCAGAGTGCTTTCTTCATACATGCTAGGCAAAAGCTCTACTA
CTGACCTGCAGCCCTAGCCAGATACATATATGCATATACACATAGATACACACACACATGTGTATTATTC
TCTCCATGTAGTCCTTGGCTGTTCTGGAACTTACTCTGCAGATCAGGCTGGCCTCAAACTCTGCCTTCCA
AGGGATGGCTTGTGCCACCATGCCTGACCAGAGATTATCTTTCCAAGCTATTTACCTTCCCTGCTTGAAA
GCGCTCTCACTCTTTCTCATTATGTTTTTAATCCCCAGTCTGGTCCAGTGTGAAAGATGCTGTTGCTCCC
TCGATTTGCAGGGAGCTTGGGGCATGGGAGCAAATGACTTACCTGAAGTAGAGTGGAGACAAGGGCGAAT
CCCCAGGGCAGCTCAGCCTAGGGGCCTTGGGACTGAAAGGACAGGAAAAGAGGAGGGACTGGGGAGCTGT
GGTGCAGGGTAGCCTGAGGGAGGGATTGTAGAGGCCAGAGGAAAAGACTAGTCTAACCCAGTGCCTAGCC
CATGAGCAGGAGCAGCACTGGGCCGTTGTTAACACACAGGAGGATTTTATCCGTAGGGATGACCCTTGGT
CTCCCAGCCCCATACAAAAGTATTTAGGGACAGAACCTCAAGGGTTAATTGTGGCAGCACAGGCTCTGGC
TCAGGACCGAGGTTTTCAACTGTGAATCCAGGCACTGGCACTGAAAGGTGGGTGGTTGTTTGTTTGTTTG
TTTGTTTTGGGTTTTTTTGTTGGTTTGTTTGTTTACTATTTCTCCTTTATATGTTAAAACCACGACTTTA
AAAAGCAATGGGGGCAGGGAGGGAATGGTTCGGTATTAAAGAGCACTGGCTGCTCTTTCAGCTCCCTGCT
CCCACATGGCAGCTCACAACCATCTGTAACTCCAGTTCCAGAAGATCTGATGCCCTCTTTTGGCATCACA
TATACACAGACATATACACAGGCAAAACACACATACACATAAAGTAAAAATAGTACATTAGTATTAGTGG
GCTGGTTAGAAGCTGAGCAGATAAAAGTATTTGTCATACAAGCCTCACTTGTCTTACTTTGATCAATGGA
GCCCACAGGAGAAGGAGAGAACCAGCCCCTGAAAGTTGTCCTCTGACCTCCACACACACTCACACACACA
TACACACGTACTCACATACACACATACACACGTACTCACACACACGCACATACACACGTACTCACACACA
CACAAACACACGTACTCACACACACACACACATACACACGTACTCACACACACACTCAAACGCACACACC
ATAGCACACATGCTGTACATTCACTCACCTCTCTGTACACACACACTATAGACATACTAATAATAAATAA
ACAATGAAGTAGTGGTGGCGCCTGCATCTTCAACTATCAGTCTATCCGAGTGGACACGATGAGTGTGCTT
AATGGCTGGTGTTGAGGAGATGGACTCCTGGCCATCTGTGCCAGCGGGAGTCCAAAATTGGGACAGTCTC
CTGGGAGAATCCTAGACAGGACTGGCCACATGGGCAAAACATGAAACCTGTTTCTCAACAATGTTGCTTC
TGTATTTGAACCCTGCAGAGCCCTTTTGGCCCCAGTATACATTTGTAACAGTAACAAATCAGGGCCTGGG
GGTGGGGAGGAGGGGAGAGGGGTCAGAGCAAAGAGCTTGCTATACTTGCAGAGGACCCAGCATCGATTCC

CAACCTCTGCATGGTGGATCACAAACACCCAGAACTCCAGTCCTAGAGAGTCTGATGCCCTCTTCTGGCC
TCCATGGGCACCAGGCATACATGACATGGTACACAAAAATACAGACAGACAAAACACTCAAGCACTACAT
GTAAAATATAATAAAATAAAATAAGGAATCAGGAACAATCTAAATTTCCTGGATGGGCCAGGAAATACAT
GGTAGCATCCATAATGCTATGCAAATCAACCAGCCATCCAACAGTGTATCTGTGTATCAATAGAGGGATG
AGTTTCCACGATACCTCTAAGTAGAGGGGAAGAAAAGGAGAAGTTATCTTCCCAAAAGCTGAACTATGTA
TCCATGTTTGAACTTGTCTTGATTATCTCCAAAAATGGAAAACAAAAACAAAAACAAAAACCTCGAGGAA
CGAGGTTGCTGGAGCTGAAGCCTGAGGCAAGCCACTTCCCTTCCAGGAGGTTCAACTCTCTCTTCCTGTG
GCTCTGAAAGAGTATAAGAGGACCTGGAGAGACCTGAGCTTACATATATACACATATACACACCTGGAGA
GACCTGAGCTTACATATATACACATATACACACCTGGAGAGACCTGAGCTTACATATATACACATGTGCA
CACACACACACTTGTACTTACACTCATATGCATACATACATACACACAGTCACATACACAAACACTTGTA
TACACTCATATATACCTATACACACTCACATACATGCACATACATATACACACGCTCACACACACAAACA
TAAGCTGAAGCAAGATGGCGGAGTTGAGCCTGGCCTTCTGCCGGCCACAGGGTGAGCTCAACCTGTCTGC
TGGTTCCTGTCCTCTCTGAAGCTAGAATTCTTTCACTGCTAAAACAGGGCGGGAGGAGTCCAGAGCCCTG
CCACTGGGTGCAGAACATGAAGACCCCTTGAGTGGAAAGGGGTTATACAGCTGGACAGTGGTGGCGCACA
CCTTTAATCCCAGCACTCGGGAGGCAGAGGCAGACGGATCTCTGAGTTCGAGGCCAGCCTGGTCTACAGA
ATGAGTTCCGGGACAGCCAGGGCTACACAGAGAAACTCTTGTCTCGAAAAACCAAAAAAAAACAAAAAAG
GAAAGGGGTTACACAACAGAGACTCAGGTCACAGCTACCCATCACACACAGGATACACATCCAAAGGTGT
TCACAGGCAGATGAGGAACGAGGAGAAGGGGCTCAAGCAAGGGCCTAAAGTTTCTTTTTTTTTTTTTTTT
CTTTTTTTTTTTCCCTGTGGCCTAGAGTTTCAAGAGGCTGAGGACCTAGGCATGAACCAAGAGGGGCCAA
ACCACTTCAAGAAGCAGGGGGTAGCAGCAGAATCTCACTATCAGCCTTGAGCACAGCTGGGAAGGAGATC
CATGGAAACAACCAAGAAAGAGCTGAAAGGGGCTGGAGAGATGGCTCAGCAGTTAAGAGCACTGAGTGCT
CTTCCGAAGGTCCTGAGTTCAAATCCCAGCAACCATATGGTGGGTCACAACCATCTGTAATGAGATCTGA
TGCCCTCTTCTGGTGTGTCTGAAGACAGCTACAGTGTACTTATGTATAATAAATAAATAAATCTTTAGAA
AGGGAGGGAGGGAGGGAGGGGGGGAGAGAGAGAGAGAGAGAGAGAGAGAGAGAGAGAGAGAGAGAGAGAG
AGAGAGAGAGAGAGAGAGAGAGCTGGAAGAGGGAGATCTGGGAAGTCTGCTGGCTTTATATGCTGACCAT
ATATAGTCACCTGTGTTTACAACTGTGCTCATCACTTTGAAATCTCAGTGGTTTCTCCTTTGAGCCTGTG
TCTGTAAGTACACCAGGACAGTGGTACATAGGCAAGAATAACAGCCAGTGGGCATAGGACACAGAGTGCA
TGGGCCCCAGCAAGATGCAGAGAGAACAGAGCTCTGGCTCCTAAGACACAGGGCC**TTCTGGGAA**ACTCAA
GCAGCCAAGCAACCCTAGCCAGCCCTTTCCTGGTGGCCCTCCTTCTGTTCCAGCAAAGGCGGAAATGGGA
ACAGGGGTGGAAGCAGAGCATTGGCAGAGCATAGGTATGACTTAGTCTTGACTAACACAAGCATGGCAGT
AGCCTGACAGTGGCCTAAATGTGGGGATGACTGCCTTAGATGAGGATGACTGCCTTAGATGGGGATGACT
GCCTTAGATGGGGATGACTGCCTTAGATGGGGATGACTGCCTTAGATGGAACAACAAACATCTATGGGCA
TGCTGTGGAACACTGGCCCACACACGGAACTGAAGCACTGGCAATTTCCACAGGGCAGTTAAACCTAAAA
GCATGCTCACACTCAACAGGCTGCCGGAACTCATGAGACACCTGGAATAGACGAATGTAGAAACAGAGCA
GAGAGGTGGTTGCCAAGGTCTGGGGGCTCAGAGGACAAGCAAGAGGCGCGGCTTTCCTTTGGGGCTGGCA
TGAAAGGAAATATCGAGGTTACAGCCTGAGAGGGCTTCCCCTGACACTTCGTATTCAAAGAGGCCATGGG
CCACCAGTGAAGACAAAGGAGTATGGCCTGCACCACAGGCTGGCGCTGACAGTCAGTAAGCACACAGTCA
CTCTGGGTCATCCCATCCCCTTCCTTGCAAGAGAAATCAAGGAAATGTCCCGAGAACAATGGGGCACAGT
GCCCAGCAGGACATCTCTTCCTGCCCATGACACCCTTGGCACAGTATGGGCCC**TTCTGGGAA**GTGGCCTT
CCAATGTGCTCTGCACAGGCAGCTCCTTTTCAATGTATGCCCGACACTCTCTACATGGAGCAAGCGCCTC
CACACTCTTAGAAGAATTTTTAGAAAACTCCAGAAAAGCACCAGGAGAAGTCACCCTCAGATGTAGCCCG
GACTCGAGCCTTGCTCAAAACCTCCTGTCTTGTTTTCTATGTGACTGTACAAATTTGGAGCTCAGAATTG
CCTTTGTCTGTGATGG~~GTTCCAACCCAACCACTCAAAGTGACACTTGTCACATTTGTCACTGATCCTATT~~
~~TCTTCTTTTTCTGCTCCTTCATTTTCTCCGCTTTCATAATAAACAAGTATTACTTTTTAAGTGGGGAAA~~
~~AAATGACCACCCTTACAAAGGACTTTTTAAAAATGGCCTCCATTGTGGCCCTTGTTCCTGGCAGCCTGGG~~
~~CCTGCTCTCTCTGTGTGGCCAAGAAGGAAGTGTTGTAGCCCATCTAGAGCTGTGCCAGCCTCTTCCCCCA~~
~~CCCCACCCCCAAAGTCTTCCTCCTGTGGGTCCTTTAAATGCATCCCAGACACTCAGACAGCC**ATC**AGTCA~~
~~CTTGCCTGACACCGGTACCATGCGTTGCCTCATCAGCCTTGTTCTTGGCCTGCTGGCCCTGGAGGTGGCC~~
~~CTCGCTCAGAACCTAGAGGAACAAGTCTTCAACTCAGGTAAGCCCCAGTTTCTCACCTTACTACCGGGTG~~
~~TGGCTTGGAGAACCACACTCACACATACTGATCCCAGCTGTCTATGCTGAGGAGGCAAGGAACAAAAGAA~~
~~CACCCCTAGAGAAGAATCCAAGGGTGGCAGGAAGTAGGGGCCTGTCCCACACAAGCTGTACAGCCAGGAA~~
~~CCTGGGTGGAAATGTTCAGGCTAAGAGACAGGTTGATGAGAGTTACCCTCAGGCCCTGGTCACTTCTGGT~~

~~CAGATTCTTAATGTTCTACCTATAATGATGAGCCTCTGACCTAATGCTCTGCCCAGGTCCATGAGCCCAT~~
~~GACCTAATGCTCTGCCCAGGACCATGAGCCCATGGCCTAATGCTCTGCCCAGGTCCATGAGCCCATGGCC~~
~~TAATGCTCTGCCAGGTCCATGAGCCCATGGCCTAATGCTCTGCCAGGTCCATGAGCCTCTGACCTAATGC~~
~~TCTGCCCAGGTCCATGAGCCTCTGACCTAATGCTCTGCCCAGGACCATGAGCCCATGGCCTAATGCCCTG~~
~~CCCAGGACCATGAGCCTGTGGCCTAATGCTCTGCCCAGGTCCATGAGCCCATGGCCTAATGCTCTGCCCA~~
~~GGTCCATGAGCCTGTGACCTAATGCTCTGCCCAGGACCATGAGCCCATGGCCTAATGCTCTGCCCAGGTC~~
~~CATGAGTCCATGGCCTAATGCTCTGCCCAGGTCCATGAGCCTGTGACCTAATGCTCTTCCCAGTTCCATG~~
~~AGCCCATGGCCTAATGCCCTGCCCAGGACCATGAGCCTGTGGCCTAATGCTCTGTCCAGGTCCATGAGCC~~
~~CATGGCCTAATGTTCTGCCCAGGTCCATGAGCCTCTGACCTAATGCTCTGCCCAGGTCCATGAGCCTCTG~~
~~ACCTAATGCTCTGCCCAGGTCCATGAGCCTCTGACCTAATGCTCTGCCAGGTCCATGAGCCTGTGGCCTA~~
~~ATGCTCTGCCCAGGTCCATGAGCCCATGATATGCTTCCTAACTCTCACAGCTACTTTCTCATTCTGGCAG~~
~~GTTTTCTGGTTTTCTTAGTTTGGCTTGGTTTGGTTTGCTTTGCTTTGGTTTGGTTTGGTTTGGTTTGTTC~~
~~ACCTTTAAGGCTCCTCAAAGGCTTGACTTTTTGTAGCTTCTATCCATGTCTCCATGCCTTCTTCTGAGAA~~
~~GCCTTAGAAGTGAGGTTGATGTCTGGGCCCCCATTTACCTCCACGTCTTTTGTGCAGTTCAGTCCATGTT~~
~~CCCAAAAGCCAGCCCCATTGAGGGCACAGAGTGTATCATCTGCCAAACCAACGAGGAGTGTGCCCAGAAT~~
~~GCCATGTGCTGTCCCGGTTCCTGTGGTAGGACCCGCAAAACTCCTGTCAACAGTAAGTCCCCGCCCAATC~~
~~CCGGTGGTGGGAGGAGCCAGCAGGGTCAAGAGTCTGCCTGGAGAGGACACATAGTCCTTTACCCACACA~~
~~CCAACTCTGTGAACCCCACTTGCCTGTCAGACCCTCCCTGCAGCCTGCTGTCCATCCTAGCCCCTGGGGT~~
~~CTTGAGCTTCCAAGGCGCTGCCTGACCATAAGACAGACTTCAACAAATGCCCCATTCATGAAATAGGGC~~
~~TTTTCAGTCATGGGGTCAGTAAAGTGCACTCAGCAAATATGGGGCATGAAAGGGCTTTGGGTGGTAAGAG~~
~~TGACCATCTCTCCCCAGTTGGTGTTCCGAAAGCTGGCTTCTGCCCTTGGAATCTACTCCAAATGATCAGT~~
~~AGTACCGGGCCGTGCCCAATGAAGATAGAATGCTCCAGCGACCGTGAGTGTTCTGGCAACATGAAATGCT~~
~~GCAACGTCGACTGTGTCATGACATGTACACCCCCAGTGCCAGGTATGCAGTCTCAAAACCCTGGACCCCC~~
~~ACTTCTCCCTAGATCTCCTCTGGCCCCCACCTTACCCTGCATCAGATCCAAGTGCAGATACAATGTGAGA~~
~~CCAGCCCAGGACTAGGTCCCAGGGTAGGAGGAGAATGTCCTAGCCAATCCTGCAAGCCTGTGGGGACTTT~~
~~CTGTGGCCACTCACTGTAGAAGTGAATGTCAATGAACAATGGACCTGAGAGAACAGACCATTCTAGCACT~~
~~CAAAACTAGTCCTGGTGAGTCTATGGGCCACATGGTGCCTTAGGCACTGAACCTGAGCAAACAGTAAAGT~~
~~GCTCAAAGTTAAGAACATGAGCTCACATCCGGCTGCCCATGTCCACCTGGGCGCCCCCTGCAGGCGGGCG~~
~~TGTCTCACCGACCTGTCTATGGGTGATGGGGGCACTGTAAAGAATTGGGCTGGAGAGATGGCTTAGCAGG~~
~~TAAGAGCACGGACTGCTCTTCCAAAGGTCCTGAGTTCAAATTCCAGCAACCACATGGTGGCTCACAACCA~~
~~TATGTAATGAGATCTGACGACCTCTTCTGGTGCGTCTGAAGACAGCTACAGTGTACTTAGGTATAAAAAT~~
~~AAATCGCCGGGCAGTGGTGGCGCACACCTTGATCCCAGTACTTGGGAGGCAGAGGCAGGCAGATTTCTGA~~
~~GTTCGAGGCCAACCTAGTCTACAGAGTGAGTTCCAGGACAGCCAGGGATACACAGAGAAACCCTGTCTTG~~
~~AAAAACCAAGAAAAAATTAAAATAAAAAATAAATAAATAAATCTTTGGGCTGGAGCGAGCAGGGACTGAG~~
~~CGAGCGGGGCTAACCAGAGCAAGCAGGGTTGACTGGAGCGAGTAGAGGTCCTAAATACAATTCCCAACAA~~
~~CCACATGAAGGCTCAACAACTGTACAACTACAGTGTACTCATACACATAAAAATAAATAAATAAATCTTA~~
~~AAAAAAGAAAGAAAGAATTGTACACACTCACCTGCAAAGCCAGTAAAGGTCTCCAGCCTTCAGGTCCCTG~~
~~GAATGCTGGGCCTGACTAGGCTAGAAGCAGAGGTGACCTCCCTCCTCTCATCCCCATCCATCTCCCCTAC~~
~~AGTGATAACCCTTCAGTGAGCAGCCGGCCCTGGCATCCCTGCCTGTCGGGAGTGACCAGCCCAAGTCTAT~~
~~ACAGCAAGACCCTTCACTCCTGGATCCAGAGAGAACATAATGCTTTCAATCTGCTGCTAATAAAAATCCA~~
~~TTTGGCTTTATGTCTCTGTCTGTCTGTCTGTCTGTCCTGAGCTCCTGAAGGAACGACAAAGCAGGCACAG~~
~~GCCTGCATTACCCTAGGATCTCCAGGGTGGCAAGACAGCCACAGGTGATTTCCCAGTAGTGAGGAGCAAG~~
~~CATGGAGGGTGGCATGCTGACTCAGGCTTGCTGAGCTACAGCCAAACTCCATCCAGATTGTGGACCAA~~
~~GGTAGACCTCAGGACCAGGAAAGAAATGGGCATCGTCTGCATCTGAATCCTTCCAAGGTACGACCAGCCC~~
~~CAGCCCCAGACAGAAGAGAAGCTGAGTTTGCAAGGGGCTTGTCTTAATTGGGGTTTCCACTGCTGCAATG~~
~~AAACACCATGACCAAAAAAGCAAGTTGGGGAGGAAAAGATTTATTTGGTTAATACATTCATGTCATTCAT~~
~~CACTGAAGGAAGTCAGGACAGGAACTCACACAGGGCAGGAACCTGGAGGCCGGAGCTGATGCAGAGGCCA~~
~~TGGAAGATGCTACTGGCTTGCTCAGCCTGCTTTCTAATAGAACCCAAGACTGTGGTCTCTGCAGCCCAGG~~
~~GATGACACCATCCACAGTGGGCTCCTCCCACCCCAATCAATAACTAGTTAAAAAAAAAAAGTCTTACAGA~~
~~TGGATCTTTTTTCTTTTTTAAACTTATTTATTTACTATATGTAAGTACACTGTAGCTGTCTCCAGACACA~~
~~CCAGAAGAGGGCATCAGATCCCATTACAGATGGTTGTGAGCCACCATGTGCTTGCTGAGATTTGAACTCA~~
~~GAAGAGCAGTCAAAGCTCTTAACCACTGAGCCATCTCTCCAGCCCCTTACAGCTGGATCTTAAGGAGGCA~~

~~TTTCTTCAATTAAGGTTCCATCCACTCAGATAACTAGTTTTATGTAAAGTTGATGGAAGACTAGCCAGCA
TTGGGAGTCAAGGCCTTGAAGCCTTAGCTAACGTGGAAGCGTGGAGGAAGCAAGGCCGTAGTAATCGAGTG
CACAGGGTAAGGTCAGCCATGCACCGACACAGCTCCCAGCCAGCATCGTATAGGCTCTCCAGCAAGGCAT
GTGTGCCCCACTCAGACAAGCAGCATAACGCACAGCTCCTGCCAATGCTGACAATCAGCCCAGATTCACA
TCAGACTCCAGCTTCAGATACCAGCTCCAAGTCTAGCGCCGCCCCACTTCCACCGTCCCTTTGTAACTAG
AAGTAGAGATGTTCTAAAGAGAGACTCATGACCCCTCCCTTTGGCTATCAGGCTACTGAGACATTCTCAC
TCTGGGAGAATCAAAGGGCGGAAGCTCTGTCCAGTCAGGAGAAATCTGTCTCTGGTGCTGGTTATTTGTT
TTTATTTTTGTTTTGTTTTTCTGTCAACTTGACCCAAGCCAAGGTCACCCAGGGAGAGGGAACCTCGTTT
GAGATTCCCTCAGATTGGCTTGTAGACATGTAGGACATTGTCTTGATTAGTCATTGGTGTGGAGAACCC
AGGTCACTGTGGGTGGTGCCATCCCTGGGCAGGTAGCCCTGTAGGTATATTAGAAAATAAGTAAATAAAA
CTAGGCGAAACAAGTCAGGGTGAGCAAGCCAGTAAAGTTCCCGCATGGTCTCTGCTTCAGTTCCTGCTCT
GAATTCCTGCCCTTCAAAATGGACTACAACTGTAACTGTAATAAGCCCTTTCCTCACCACAAGTAGCTTT
TGATCATGGTCATTATCACAGCAATGCAAACTTCCCAAGAGGAGAGTTCACATATGCTGACAGCTTACCA
GACCACCTTACCTTTGGATAGATTGGAGAAGAGACAATGTCAGGTCAGTGATGGGCGGGACCACATCTTG
CCCAGGGCTGCTCAGCTGTGTATGCCTCTTGTCCCCTATGTAAGTCTAAACCTCACCCAGGACCTCAAAG
ATGGAGCTAGGGAATCAATGGGCCTCTTTATTTGTTCCTCAGCTTGTAACTACTAAATGATCGTTAATTG
GTGTGCTGAGGGCAGGTGGCTCAGCCTGGCTTGTTAGGACTGCCAGAGCCCAGGCCTTGACACTAATTAC
TCCAGAAATACCTTCAAGTTAAATAAGCCGTTAGAATAACTCATAAAAGCACTTCAAGCACTGAGCTTAT
AAAGGGTCATGAAGGACAGAGATCAAGAGTAGCCAGATTGTCTTACTCGATGACCTATGGCCATAAAGAG
ACACCATGGCCATGACAACTCTTATAAGATTTTAATTTAATTTAATTGGGGCTTACATTTAATTGGCTTA
TATTTTCAGAGGTTTGGTCCATCATCATCAAGATGGAAGCCTGATGACACATAGGCAGATGTGGTGCTGG
AAAAGGAGCTGCAAGTTCTACATCAGAGCCCACAGGCAGCATGAAGAGAGGGTGCCACTGGCCCTGGCTT
GGGCTTTTGAAATCCCAAAGGCCACCCCTAGTAACACATTTCTTCCAACAATGAGGCTATACCTCCCAAT
CCCTAATGGCCACACATTTAAATCTTTGAGCCTAAGGGGACCACTCTTATTCAAAACCCCACACAGATGA
AGAGAAGTAAGGAGGGGCCAAGGGGTGGAAGATGCAGGAATGTCCCTATGGGACAGACACCCACCAGGCA
CACCAGAAATCTTCCTCATCATTCAAGTCCAGAGCTTTTAGGAAGTTTCATTACATAATCATGATCGATT
AAACTACTGACTGTCGGTGACTGAATTCAGCCACCATCCCCTCTCTCGTACCTGGAGGTCATGAGTTGAT
CTGGGAGGTCCAGCCCACACTAATCCTGTGCCTGGTCTTTGTGGTGACCAGCCCATCTGAACCTGCAGAA
GTTCCCATATGATCAGTCCCCATTTGAAGCTATGGAAACCACCCTTTCAAGTCTCTATGCTGGAAGATA
AACATCTTGTCACACAAAGAAGCACATGAAAAAAGCAAAAGGAAGTCAGCACGGGCAAGGCAATTTCTTT
TCTGCAAAAAGAAGCATGCATCAGAAGCCAAACCAGGCTAGAAAGCAGCACTCTTAGCCAAAATGGCCTC
CAGCTCTCATCTCTGATGTAAATGGTGACTGTGTTTCTTCCCGTTGGTGGAGCTCAACTTCATAACTGA
TGGGATTGACATTTGACGCAATTAGCTAATGGGTATAAAGAGGGAGGAGGGGACTTGGGAAATATGGGTC
CTTGTTAGGCTAGCTACCTGGGTAGAAAAACACAAAACAATATTTCTTACACCCATCTGAAGCTATGATT
TGGCTGCATCTCAAATGAACTGGATCAGACTAGGGTAGTGTTTTATAGCAATTTCCCCTGAGACAAAAAT
CCTCCATCTTGGAGAGAATCCTAGAAAGTCATGGGATTTTCTAAAAAGGGGTGCCAGCTAAAACTCAAAA
GCTCCATCCTGCAGAGAAGCTAGTTAAAACTCAGGAAGTAAACAACACAAAACTTTCAGGAAGTTTCTGA
AACTGATCACATGTGCTAGGCTCCTCCTTCCTCAAATGTATATAAGCAGTAAAAACTGCTGAAAGACACT
CTTGGGCCAGTAAGCCATATGGAAAAAAACTCAGACCAGCAAGCTACTTGTAAAGGACCCTCCCCAGCCC
ATGGAGCTGCCTACCTGCTGCACAGTATGCTCCAGGTTCCCAGTTTTCATGAGCCGTTGCTCACACTAGG
GTGGGCTTCGGTAGTTAAGTCGTCTCGAGTCACTTCTGCTCCTGTAAGTAACCCTCTGTGGTGGTGTGAA
TGAGAACGGCCCCATAGGTTCCTGTGCTTGGATGCATGGTCATCAGCCGGTGGAACTCTTTGGGAAGGAC
TAGGGGCTGTGACCTTTTGGAGATGTGTCACCAAGGTTTCAAAAGTCCATGCTATTCCCAGTGTAGGTTC
TTTCTCTCTCTGCCTCCTACTGTGGATCAAGATGCAAACCTTCAGCTACTGCTTCAGTGCCAAGCCTGCT
TACTTCCATGCTCCTTGTCATGATGGTCATGGACTTTAACCCTCTGAAACAGTAAACTCTCAATAAACTC
TTTCTGTATGTTGTCTTGATCATAGTGGCTTATCACCGCAATAGGAAAGTAACTAAGACACCTTCACTCA
TACTGCTATGAGTAACCCAAAGAACACTCATTGTTTCATCAAGCTGGACTTTGGCAGTCTCTTACTGTGA
TCCATCATTGGTTCCCTATCTGGGCTGAGTAGATATTTGTTCATGTCTCTCCTTTCTTGCCTCCTACCAG
TCATAATAAAGGGAAGGTCACATGGCTTTGCCATCTTAAGGGCCAAATACTGGGAAGATTAATTTTAATT
GTTAATTTGACTGAATTTAGAATCAGCATGGAGGAGCTGGAGAGATGGCTCAGTGGTTAAGAGCACTGGT
TGTTCTTCCAGGAGTCTGAGTTCAATTCCCAGCAACCACATGGTGGCTCACAACCATCTATAATGGAATC
TGATGCCCTCTTCTGATGTGTCTGAAGACCGTGACAGTATACTCACATATATAAAATAAATAAATCATTA~~

~~GAAAAAAAAAGATACCAGGCAGTGGTGGCCTTTAATCCCAGCACTTGGGAGGCAAAGGTGTGCTGATTAA~~
~~GATGTTTCTAGACTGCTTGCTGACAGGAGCGTGATAGAGCTGTCTCATGAGAGGCTCTGCCAGTGCCTGA~~
~~CAAATACACAAGTGGATGCTCACAGCCTTCCATTGGACTGAGCACAGGGTCCCCAATGAAGGAGCTAGAG~~
~~AAAGGATCCAAGGAGCTGAAGGGGTTTGCAGCCCCATAGGAGGAACAATATGAACCAACCAGTACCCCCA~~
~~GAGCTCCCTGGGACTAAACCACCAACCAAAGAGTACACATGGTGGGACTCATGGCTCCAGCTGCATATGT~~
~~GTCAGAGGATGGCCTAGTCAGTCATCAATGGGAGGAGAGGCCCCTTAGTCCTGTGAGGACTCTATGCCCCA~~
~~GTATAGGGAAATGCCAGGGCCAGGAAGTGGGTATGGGTAGGTTTGTGAGCAGGGGGAAGGGGAATGGGAT~~
~~AGGGGGTTTGGGGAGGGAAAATCAGGAAAGGGGATATTAACATTTGAAATGTAAATAAAGGAAACATCCA~~
~~ATAAAAAATAATTGTTCTGGGCTGGTGAGATGGCTCAGCAGTTAAGAGAACTGACTGCTCTTCCAGAGGT~~
~~CCTGAGTTCAATTCCCAGCAACCACATGGTGGCTTACAACCATCTGTAATGAGATCTGATGCCCTCTTCT~~
~~GTTTGAAGACAGCTACAGTGTACTCACATACATAAAATAAGTAAATAATTTTTAAAAGGAAGGAAGGAAA~~
~~GAAAGAAGGAAGAGAAATAATTTCACTTCCATTTGGGACTCAGAAAAAGTATAGATATCTGTCAAATAGA~~
~~AAAACACAAATTTGGTAAAAGAACAAAAAAAAAAATTGTTCTTCTCATACACTGAAAAAAAAAAAGATGTT~~
~~TCTAGATGACTTTAACAAGGAGAAAAGACCCATGTGAATGGGAGGGAAGGGGTGGAGAACACCATTCTGG~~
~~GGACTGGAATACCAAAATAAATAAAGAGAAGAAAGTCAAAGCCAGGCATGGTGGCACACATATTATTATT~~
~~ATTATTATTATTATTATTAATTTAAAGTATGACTGCTCTGATGCATATACACCCTCAGGCCAGAAGAGGG~~
~~CACCAGTTCCCTTATAGATGGTTGTGAGCCACCATGTAGTTGCAGGAATTGAACTTAGGACCTGTGGAAG~~
~~AGCAGCCAGTTCTCTTAACCTCTGAGCCATCTCACACACATCTTTAATTACATCATGCAAAAGGCAGAGG~~
~~CAGGAGGATCTCTGAGTCTGAGGCCAGCCAGTGAGCTCCAGGGCAGCCAGGACTGCTCTAAAGACTGTCT~~
~~CTAACCCTCTCCCCCCACCCTGCAAAAAAAAAAATAAAAAATATAGCTGACCATCAACATTCATTTCTCTC~~
~~TGCTTCCTGACTATGCCTTTTGGGTCTGAGGTGAGGAGGTGCAGTAGCTCTGCTGGCTACTGTGTGCTCA~~
~~GGTGGCTCAGCAGGTCACCTGTGCATGGAGCTCAAGGCCAGTTCAGTTTCAGCATCCACACCATTCACGT~~
~~TAGGACCAGTGGTCCTTCTTCCATTCCCTCAAGTCACTGCAGGACTAGGCACCTCCTCCCCACTGAGGCC~~
~~AGATGAGGCAACCCAGTTAGGGGAACAATATCCACAGGCAGGCAACAGAGTCAGGGTAAGCCTTAAGAAG~~
~~CATTCTTGGGGCTGGGGAGATGGCTCAGTGGTTAAGAGCACCGACTGCTCTTCCAGAGGTCCTGAGTTCA~~
~~ATTCCCAGCAACCACATGGTGGCTCTCAACCATCTGTAATGGAGTTTGATGCCCTCTTCTGGTGTGTCTG~~
~~AAGACAACTACAGTGTACTCATATACATTAAATAAATAAATCTTAAAAAAAAAAAGCATTCTTTACAGCA~~
~~CGTTGGTCAATACAATGCCCATGTCCAGATTACAACCTGATCCTGGTCACAGAACTCAGCAGCATCTAAG~~
~~CATATCTTGCACAAGACCAACCCTAGCAATGGCCTCTGCTCCAGTTTCTGCCTTGAGCTCCTGTCCTTTC~~
~~TTTGATGATGATGTGTGATGTGAGTCAAGTAAACCACAAGTTGCTTTGGCTATGGTTTTTATCACAGTGA~~
~~TAGCAACCTAACAAAGACATTGGCCAAAGTCCATCTTAAATCCCCTAACACTTTGGGGTTCCTTGCTCCT~~
~~TTCCTAAAGCTCTACTCCCCAGCACAAGGTTGCCTGACAAACATGGTGGCTGACCCTATCTGAGCTGCAT~~
~~GGCTCTAGCTCCCGGCCACATGGATGCCTGCCACCCGGTGGCTAACCTGTGAACTGCCCAGCTCAAGCTG~~
~~CAGGGCTATTCTCCAGTCCCCAGGCAGCTTTGAGGTTTATGGCTGACCGGCCCCGGGGTTTTCTTTTTGA~~
~~ACTCTAACTGTCCTAGGCTTCCATTTGTGTGTATGTCCTAAATTATTTTATTAATAACCCTAAGGAAGAA~~
~~CCCTGATTCCCTAGGCGCCATAGAATGGCTCTCCTGGGGTTCCCCAAGATCCCCAATAACACATCTTTCC~~
~~TACTTAATTATTTAATTGGCAGAGAAAATGAACCCATTCCCATACTTCTAGCTGCCATAGTCCTGGGGCT~~
~~ACAAGGCCTCTGGCACCTGGTCTAGCCTGGGGTTGGTTCCAGGAACTGTGGCGAGGTATAGGCATGAGAT~~
~~GAGGGTGGCTCACAGCTTTAAGGAGCCTTGAGTGGAGGTGCTGTCTTCAGAGGCTCCAGCCTTTCACTGT~~
~~TACAGAATATCTGATCACACTGTGAACCCTAAGACTGTGTTACTTACTGGAAAACCTGTTTGTAGTTGTG~~
~~GCAGCTCTTAGCACACAGCTCTAATCCCAAACAATGACAGTAAAGTAAGTTTGTAGAAGGAAGCACCCAT~~
~~GTTTGAAAGTGATGTCTACCTAATTGAGTGGCAGGTGAAGTGAAGAATCAGAGAAGGATCTGACAGAATA~~
~~TGATCTGCCCAACTCTCACAAGAGGAAAGGGAAGCTACTTGAGGGGCAGCGCAGAGAAAAGGAAGGAAGC~~
~~GGTTTTACTGCGACAGTTGTACAGAGGCAGATTGCCGAGTGAATAAATTAGACACAAGTGAAGACAGGAG~~
~~CCAGAGAATGAGGACCCAGAAGATTACAACAGATTGCCAAAGTTAGTAGGAGTTCAAGCAAAACAGTCAA~~
~~GAGGCCAAGAAAGAAGCCAGCTTGAATCAGTCAGTGTGGAGAGTGTGGAGAGTGTGGAGAGGAGTTTTGA~~
~~GCCAGAACAGCTGAGTTGACCAGCTCAAAAGAAGCTAGAAAGGGTGAGCTTATTCAGCAACAAGTCTCAG~~
~~AGGCTGAAAACATTCTAGGCCTAGATAAGATTCTACAGAGGCTAGAAGCTTCCAGGACTAGGCCTATTTA~~
~~GCAAACAGAGGCAGTAGGCCTCCAAGACAACAGTCCCAGCACTAAGAATTTTATACATTCTCAGCTGGCT~~
~~GTGGTGGTATGTCCACCACTGCAACCCTTCATTTCCTTTCTGGTCCATTTTATCTTTGTTTTGTTTGTTT~~
~~GTTTTTCCTTTTTGTCATTTTTGCTTTTGGTTTCCTACTAAGAAACTACTGTTTAATCCAAGATAGTAAA~~
~~AATGTGTTTCCCCTTAGGAGTTTTATATCTATATTATCTCTGACCTTTTTGAGCTAAATTTTGAATTCAG~~

~~CATGAGACTCTTAAACAAGAACCACCAAGGTAAAGCTAGTGTGGCAATATAATGCTAGCTACTGTAAGGC~~
~~TAAAGCAAGAGAAGCTTCCCTGGATAATTCAGCAATCTTTTATTAACAAGAACCAGCGTTTACGCTTGAT~~
~~TTTAGCCACAAAGGAGAGAAGTAATACACTCTTACAAACATAAAGATTTTTCAAGGAAAATTTTAAGTTA~~
~~AATTTTCCTTTCTACAAAATTAGGGCCAGCAGGATAACTTAGTGGGACCTGGGGGTGGGGGTGGGGCAT~~
~~GTCTTTTGTCCTGCACTCCAGAGGCAGAAGGAGGGGGATCTC~~TGTGAGTTCAAGACCAGCCTGGTCTACA
CAGCGAGTTCCAAAACAGCCAAGGCTACAAGGAGAAACGCTGTCTAGGAAAACAGACAGTTAGTTTAGGG
GCCACTTGACTGTGAGTTCAATTCCCAGATCCCACATGATTGGAAGAAACAAATTTCCCCAAGTTTTCCT
GTGATCTCTTCATGCACACATACACCTTTGTCCCAGAGATGCCGTCTGTGGTTTCCTAGCACTGGGATTA
CTGGGAAAACTCACCTCCCAACCATCCTACCCAGCATTTTGTACTATCTCCTCAGTCCTGCCGTGCGCTA
AGGCAGGGCCTTCTCCAGGTCGTCCCGGCTGTCCTAAACTTGGTATGTAGACCAAGCTAGCCACGACCTC
AAAGATCCTTTTTCCTGTCTCCTGGCTGGGGTTTTAAGGTGTGTGCCACCACGCCCGGCTATTCTGCTTC
TTGATGCAGGGCCTCCACAGTAGTACAGGCTAGCCCTCGGCCTCCTGCCTCCATTACTGAGATTACAGCA
ATTTATCGGTGTGCCGTTGTATACGGTATGTCCTTTACTTGTTTAATCTAGACAAACTGCTTGCATTTCC
GAAAAGACCTTCAGCTGGAGCTACAACTCGCTTCTCTGTAATTACCCGACACCTGGCAATGCCTTCCCGC
CCCGAACCCCAAAGTCAACACGGCGGTCCCAACTCACGCCTCTCCTACAGGACCTGTGAAAGGCCCAAGG
GAGTGGGGCAGAACGGGCTGACATCAGAGTCGCTACCCAAACTCCGCACAAGTAGGAGACTCTGGCCCGA
CGTGCCCTGCCGTGTAGAATTCACCCTCTCCTTCCCGTCTACGTTTCCCACGACCAGGATCTCACTACAC
TTCCCAGTACCGGAAGCCTGAGCGCAACAAAGATGGCGACTGCGGAAGGGGCGGAGGGCGTGGCCTCCGT
CCGGGAGCACTGGGCCACCTAGCTTCCGGCGGCGCCAGTGAC**GTC**AGTGAGCGCGCCAGGCCGCGTGG
AGGCCGTAGGCTGATGGAGGTCAGTGAGGGTGATCGCCGCCGCAGTCTGGCGTCCTGGAGAGACCGGGTT
TCTGAACGTGGGTATCCGAGACGGGAAGCGTGGCTGGGAAGGGCAGCAGTTAGTAAGGACCGGCGGGGCT
GTTGTGGGGGGAGCCAGGAAAGGGACTCTCGACACCGTGCCCCAAGGGCGCTGTTCGGTTGCAAATTCGG
TTCC

Wap-Tbrg4 (Δ12,579)

TCAGGTTTTTAGGGAAAATTCCAGCACAGCTGACCCCATAGGACAAAAAGTGCAGTGTGTGTACTACCAA
AACCCTGGTGTCCTTTTCCCACCCTAGCCGAGCAGAGTTGATGGGGCAGGAAAGAGCCTACGCATACTGG
AAGCACACAGGCTCAAGACTGGCAGGCCAAAGAACCAGAACACCCCAGGGCATAAGAACCCCATGCCCCT
TGCCGCTGGGCCTGGTGAATTCAGAGTAATGTCTTCA**TTCCTAGAA**CCTTCTGGCTTCCCGACCTGGCCT
GCAGGCTCTGAGGAGATGTGTGCACCTCATGAACTCCTTGTTCAGCCAGGGCCTCTCTGTCCTCCCTACA
CTTCCCCACCACACAGGAACACATGTCCTCAACTGACCAGTGTCACCCTGGGCCTCAAGGCTAGGTTCCC
TTGAGTACTGGGACACAAGAACAGACTTCTGCTCTCCCCTCTGTCCACACAGAGTGCAGAGAGGAAGGGT
TCTGGTTCATGTCCCACAGTTGCCCCCTAAAACCGATGTGATATAGCCCTCACTGGCCTAGAGCTCACTA
CATAAACCAGGCTGGCCTGGAACCCACAGATCTGTCTGCCTTTGACTCCTACTGCTGGGATCAAAGGTGT
GAGCCACCATGCAACGCTCTGAAACTGATTCTTTAGAAGCTAAGAAAATGCTTCAAAATGGCAGTAGCCT
TGGGCTGGAGATGGGTTAGTGGTTAAGAGAACTGACTGCTCTTCCAGAGAGAGAGAGAGAGAGAGAGAGA
GAGAGAGAGAGAGAATGAATGAATGAATCCAGAGTGCTTTCTTCATACATGCTAGGCAAAAGCTCTACTA
CTGACCTGCAGCCCTAGCCAGATACATATATGCATATACACATAGATACACACACACATGTGTATTATTC
TCTCCATGTAGTCCTTGGCTGTTCTGGAACTTACTCTGCAGATCAGGCTGGCCTCAAACTCTGCCTTCCA
AGGGATGGCTTGTGCCACCATGCCTGACCAGAGATTATCTTTCCAAGCTATTTACCTTCCCTGCTTGAAA
GCGCTCTCACTCTTTCTCATTATGTTTTTAATCCCCAGTCTGGTCCAGTGTGAAAGATGCTGTTGCTCCC
TCGATTTGCAGGGAGCTTGGGGCATGGGAGCAAATGACTTACCTGAAGTAGAGTGGAGACAAGGGCGAAT
CCCCAGGGCAGCTCAGCCTAGGGGCCTTGGGACTGAAAGGACAGGAAAAGAGGAGGGACTGGGGAGCTGT
GGTGCAGGGTAGCCTGAGGGAGGGATTGTAGAGGCCAGAGGAAAAGACTAGTCTAACCCAGTGCCTAGCC
CATGAGCAGGAGCAGCACTGGGCCGTTGTTAACACACAGGAGGATTTTATCCGTAGGGATGACCCTTGGT
CTCCCAGCCCCATACAAAAGTATTTAGGGACAGAACCTCAAGGGTTAATTGTGGCAGCACAGGCTCTGGC
TCAGGACCGAGGTTTTCAACTGTGAATCCAGGCACTGGCACTGAAAGGTGGGTGGTTGTTTGTTTGTTTG
TTTGTTTTGGGTTTTTTTGTTGGTTTGTTTGTTTACTATTTCTCCTTTATATGTTAAAACCACGACTTTA
AAAAGCAATGGGGGCAGGGAGGGAATGGTTCGGTATTAAAGAGCACTGGCTGCTCTTTCAGCTCCCTGCT
CCCACATGGCAGCTCACAACCATCTGTAACTCCAGTTCCAGAAGATCTGATGCCCTCTTTTGGCATCACA

TATACACAGACATATACACAGGCAAAACACACATACACATAAAGTAAAAATAGTACATTAGTATTAGTGG
GCTGGTTAGAAGCTGAGCAGATAAAAGTATTTGTCATACAAGCCTCACTTGTCTTACTTTGATCAATGGA
GCCCACAGGAGAAGGAGAGAACCAGCCCCTGAAAGTTGTCCTCTGACCTCCACACACACTCACACACACA
TACACACGTACTCACATACACACATACACACGTACTCACACACACGCACATACACACGTACTCACACACA
CACAAACACACGTACTCACACACACACACACATACACACGTACTCACACACACACTCAAACGCACACACC
ATAGCACACATGCTGTACATTCACTCACCTCTCTGTACACACACACTATAGACATACTAATAATAAATAA
ACAATGAAGTAGTGGTGGCGCCTGCATCTTCAACTATCAGTCTATCCGAGTGGACACGATGAGTGTGCTT
AATGGCTGGTGTTGAGGAGATGGACTCCTGGCCATCTGTGCCAGCGGGAGTCCAAAATTGGACAGTCTC
CTGGGAGAATCCTAGACAGGACTGGCCACATGGGCAAAACATGAAACCTGTTTCTCAACAATGTTGCTTC
TGTATTTGAACCCTGCAGAGCCCTTTTGGCCCCAGTATACATTTGTAACAGTAACAAATCAGGGCCTGGG
GGTGGGGAGGAGGGGAGAGGGGTCAGAGCAAAGAGCTTGCTATACTTGCAGAGGACCCAGCATCGATTCC
CAACCTCTGCATGGTGGATCACAAACACCCAGAACTCCAGTCCTAGAGAGTCTGATGCCCTCTTCTGGCC
TCCATGGGCACCAGGCATACATGACATGGTACACAAAAATACAGACAGACAAAACACTCAAGCACTACAT
GTAAAATATAATAAAATAAAATAAGGAATCAGGAACAATCTAAATTTCCTGGATGGGCCAGGAAATACAT
GGTAGCATCCATAATGCTATGCAAATCAACCAGCCATCCAACAGTGTATCTGTGTATCAATAGAGGGATG
AGTTTCCACGATACCTCTAAGTAGAGGGGAAGAAAAGGAGAAGTTATCTTCCCAAAAGCTGAACTATGTA
TCCATGTTTGAACTTGTCTTGATTATCTCCAAAAATGGAAAACAAAAACAAAAACAAAAACCTCGAGGAA
CGAGGTTGCTGGAGCTGAAGCCTGAGGCAAGCCACTTCCCTTCCAGGAGGTTCAACTCTCTCTTCCTGTG
GCTCTGAAAGAGTATAAGAGGACCTGGAGAGACCTGAGCTTACATATATACACATATACACACCTGGAGA
GACCTGAGCTTACATATATACACATATACACACCTGGAGAGACCTGAGCTTACATATATACACATGTGCA
CACACACACACTTGTACTTACACTCATATGCATACATACATACACACAGTCACATACACAAACACTTGTA
TACACTCATATATACCTATACACACTCACATACATGCACATACATATACACACGCTCACACACACAAACA
TAAGCTGAAGCAAGATGGCGGAGTTGAGCCTGGCCTTCTGCCGGCCACAGGGTGAGCTCAACCTGTCTGC
TGGTTCCTGTCCTCTCTGAAGCTAGAATTCTTTCACTGCTAAAACAGGGCGGGAGGAGTCCAGAGCCCTG
CCACTGGGTGCAGAACATGAAGACCCCTTGAGTGGAAAGGGGTTATACAGCTGGACAGTGGTGGCGCACA
CCTTTAATCCCAGCACTCGGGAGGCAGAGGCAGACGGATCTCTGAGTTCGAGGCCAGCCTGGTCTACAGA
ATGAGTTCCGGGACAGCCAGGGCTACACAGAGAAACTCTTGTCTCGAAAAACCAAAAAAAAACAAAAAAG
GAAAGGGGTTACACAACAGAGACTCAGGTCACAGCTACCCATCACACACAGGATACACATCCAAAGGTGT
TCACAGGCAGATGAGGAACGAGGAGAAGGGGCTCAAGCAAGGGCCTAAAGTTTCTTTTTTTTTTTTTTTT
CTTTTTTTTTTTCCCTGTGGCCTAGAGTTTCAAGAGGCTGAGGACCTAGGCATGAACCAAGAGGGGCCAA
ACCACTTCAAGAAGCAGGGGGTAGCAGCAGAATCTCACTATCAGCCTTGAGCACAGCTGGGAAGGAGATC
CATGGAAACAACCAAGAAAGAGCTGAAAGGGGCTGGAGAGATGGCTCAGCAGTTAAGAGCACTGAGTGCT
CTTCCGAAGGTCCTGAGTTCAAATCCCAGCAACCATATGGTGGGTCACAACCATCTGTAATGAGATCTGA
TGCCCTCTTCTGGTGTGTCTGAAGACAGCTACAGTGTACTTATGTATAATAAATAAATAAATCTTTAGAA
AGGGAGGGAGGGAGGGAGGGGGGGAGAGAGAGAGAGAGAGAGAGAGAGAGAGAGAGAGAGAGAGAGAGAG
AGAGAGAGAGAGAGAGAGAGAGCTGGAAGAGGGAGATCTGGGAAGTCTGCTGGCTTTATATGCTGACCAT
ATATAGTCACCTGTGTTTACAACTGTGCTCATCACTTTGAAATCTCAGTGGTTTCTCCTTTGAGCCTGTG
TCTGTAAGTACACCAGGACAGTGGTACATAGGCAAGAATAACAGCCAGTGGGCATAGGACACAGAGTGCA
TGGGCCCCAGCAAGATGCAGAGAGAACAGAGCTCTGGCTCCTAAGACACAGGGCC**TTCTGGGAA**ACTCAA
GCAGCCAAGCAACCCTAGCCAGCCCTTTCCTGGTGGCCCTCCTTCTGTTCCAGCAAAGGCGGAAATGGGA
ACAGGGGTGGAAGCAGAGCATTGGCAGAGCATAGGTATGACTTAGTCTTGACTAACACAAGCATGGCAGT
AGCCTGACAGTGGCCTAAATGTGGGGATGACTGCCTTAGATGAGGATGACTGCCTTAGATGGGGATGACT
GCCTTAGATGGGGATGACTGCCTTAGATGGGGATGACTGCCTTAGATGGAACAACAAACATCTATGGGCA
TGCTGTGGAACACTGGCCCACACACGGAACTGAAGCACTGGCAATTTCCACAGGGCAGTTAAACCTAAAA
GCATGCTCACACTCAACAGGCTGCCGGAACTCATGAGACACCTGGAATAGACGAATGTAGAAACAGAGCA
GAGAGGTGGTTGCCAAGGTCTGGGGGCTCAGAGGACAAGCAAGAGGCGCGGCTTTCCTTTGGGGCTGGCA
TGAAAGGAAATATCGAGGTTACAGCCTGAGAGGGCTTCCCCTGACACTTCGTATTCAAAGAGGCCATGGG
CCACCAGTGAAGACAAAGGAGTATGGCCTGCACCACAGGCTGGCGCTGACAGTCAGTAAGCACACAGTCA
CTCTGGGTCATCCCATCCCCTTCCTTGCAAGAGAAATCAAGGAAATGTCCCGAGAACAATGGGGCACAGT
GCCCAGCAGGACATCTCTTCCTGCCCATGACACCCTTGGCACAGTATGGCCC**TTCTGGGAA**GTGGCCTT
CCAATGTGCTCTGCACAGGCAGCTCCTTTTCAATGTATGCCCGACACTCTCTACATGGAGCAAGCGCCTC
CACACTCTTAGAAGAATTTTTAGAAAACTCCAGAAAAGCACCAGGAGAAGTCACCCTCAGATGTAGCCCG

GACTCGAGCCTTGCTCAAAACCTCCTGTCTTGTTTTCTATGTGACTGTACAAATTTGGAGCTCAGAATTG
CCTTTGTCTGTGATGGGTTCCAACCCAACCACTCAAAGTGACACTTGTCACATTTGTCACTGATCCTATT
TCTTCTTTTTCTGCTCCTTCATTTTCTCCGCTTTCATAATAAACAAGTATTACTTTTTAAGTGGGGGAAA
AAATGACCACCCTTACAAAGGACTTTTTAAAAATGGCCTCCATTGTGGCCCTTGTTCCTGGCAGCCTGGG
CCTGCTCTCTCTGTGTGGCCAAGAAGGAAGTGTTGTAGCCCATCTAGAGCTGTGCCAGCCTCTTCCCCCA
CCCCACCCCCAAAGTCTTCCTCCTGTGGGTCCTTTAAATGCATCCCAGACACTCAGACAGCC**ATC**AGTCA
CTTGCCTGAC~~ACCGGTACCATGCGTTGCCTCATCAGCCTTGTTCTTGGCCTGCTGGCCCTGGAGGTGGCC~~
~~CTCGCTCAGAACCTAGAGGAACAAGTCTTCAACTCAGGTAAGCCCCAGTTTCTCACCTTACTACCGGTG~~
~~TGGCTTGGAGAACCACACTCACACATACTGATCCCAGCTGTCTATGCTGAGGAGGCAAGGAACAAAAGAA~~
~~CACCCCTAGAGAAGAATCCAAGGGTGGCAGGAAGTAGGGGCCTGTCCCACACAAGCTGTACAGCCAGGAA~~
~~CCTGGGTGGAAATGTTCAGGCTAAGAGACAGGTTGATGAGAGTTACCCTCAGGCCCTGGTCACTTCTGGT~~
~~CAGATTCTTAATGTTCTACCTATAATGATGAGCCTCTGACCTAATGCTCTGCCCAGGTCCATGAGCCCAT~~
~~GACCTAATGCTCTGCCCAGGACCATGAGCCCATGGCCTAATGCTCTGCCCAGGTCCATGAGCCCATGGCC~~
~~TAATGCTCTGCCAGGTCCATGAGCCCATGGCCTAATGCTCTGCCAGGTCCATGAGCCTCTGACCTAATGC~~
~~TCTGCCCAGGTCCATGAGCCTCTGACCTAATGCTCTGCCCAGGACCATGAGCCCATGGCCTAATGCCCTG~~
~~CCCAGGACCATGAGCCTGTGGCCTAATGCTCTGCCCAGGTCCATGAGCCCATGGCCTAATGCTCTGCCCA~~
~~GGTCCATGAGCCTGTGACCTAATGCTCTGCCCAGGACCATGAGCCCATGGCCTAATGCTCTGCCCAGGTC~~
~~CATGAGTCCATGGCCTAATGCTCTGCCCAGGTCCATGAGCCTGTGACCTAATGCTCTTCCCAGTTCCATG~~
~~AGCCCATGGCCTAATGCCCTGCCCAGGACCATGAGCCTGTGGCCTAATGCTCTGTCCAGGTCCATGAGCC~~
~~CATGGCCTAATGTTCTGCCCAGGTCCATGAGCCTCTGACCTAATGCTCTGCCCAGGTCCATGAGCCTCTG~~
~~ACCTAATGCTCTGCCCAGGTCCATGAGCCTCTGACCTAATGCTCTGCCAGGTCCATGAGCCTGTGGCCTA~~
~~ATGCTCTGCCCAGGTCCATGAGCCCATGATATGCTTCCTAACTCTCACAGCTACTTTCTCATTCTGGCAG~~
~~GTTTTCTGGTTTTCTTAGTTTGGCTTGGTTTGGTTTGCTTTGCTTTGGTTTGGTTTGGTTTGGTTTGTTC~~
~~ACCTTTAAGGCTCCTCAAAGGCTTGACTTTTTGTAGCTTCTATCCATGTCTCCATGCCTTCTTCTGAGAA~~
~~GCCTTAGAAGTGAGGTTGATGTCTGGGCCCCCATTTACCTCCACGTCTTTTGTGCAGTTCAGTCCATGTT~~
~~CCCAAAAGCCAGCCCCATTGAGGGCACAGAGTGTATCATCTGCCAAACCAACGAGGAGTGTGCCCAGAAT~~
~~GCCATGTGCTGTCCCGGTTCCTGTGGTAGGACCCGCAAAACTCCTGTCAACAGTAAGTCCCCGCCCAATC~~
~~CCGGTGGTGGGAGGAGCCAGCAGGGTCAAGAGTCTGCCTGGAGAGGACACATAGTCCTTTACCCACACA~~
~~CCAACTCTGTGAACCCCACTTGCCTGTCAGACCCTCCCTGCAGCCTGCTGTCCATCCTAGCCCCTGGGGT~~
~~CTTGAGCTTCCAAGGGCGCTGCCTGACCATAAGACAGACTTCAACAAATGCCCCATTCATGAAATAGGGC~~
~~TTTTCAGTCATGGGGTCAGTAAAGTGCACTCAGCAAATATGGGGCATGAAAGGGCTTTGGGTGGTAAGAG~~
~~TGACCATCTCTCCCCAGTTGGTGTTCCGAAAGCTGGCTTCTGCCCTTGGAATCTACTCCAAATGATCAGT~~
~~AGTACCGGGCCGTGCCCAATGAAGATAGAATGCTCCAGCGACCGTGAGTGTTCTGGCAACATGAAATGCT~~
~~GCAACGTCGACTGTGTCATGACATGTACACCCCCAGTGCCAGGTATGCAGTCTCAAAACCCTGGACCCCC~~
~~ACTTCTCCCTAGATCTCCTCTGGCCCCCACCTTACCCTGCATCAGATCCAAGTGCAGATACAATGTGAGA~~
~~CCAGCCCAGGACTAGGTCCCAGGGTAGGAGGAGAATGTCCTAGCCAATCCTGCAAGCCTGTGGGGACTTT~~
~~CTGTGGCCACTCACTGTAGAAGTGAATGTCAATGAACAATGGACCTGAGAGAACAGACCATTCTAGCACT~~
~~CAAAACTAGTCCTGGTGAGTCTATGGGCCACATGGTGCCTTAGGCACTGAACCTGAGCAAACAGTAAAGT~~
~~GCTCAAAGTTAAGAACATGAGCTCACATCCGGCTGCCCATGTCCACCTGGGCGCCCCCTGCAGGCGGGCG~~
~~TGTCTCACCGACCTGTCTATGGGTGATGGGGGCACTGTAAAGAATTGGGCTGGAGAGATGGCTTAGCAGG~~
~~TAAGAGCACGGACTGCTCTTCCAAAGGTCCTGAGTTCAAATTCCAGCAACCACATGGTGGCTCACAACCA~~
~~TATGTAATGAGATCTGACGACCTCTTCTGGTGCGTCTGAAGACAGCTACAGTGTACTTAGGTATAAAAAT~~
~~AAATCGCCGGGCAGTGGTGGCGCACACCTTGATCCCAGTACTTGGGAGGCAGAGGCAGGCAGATTTCTGA~~
~~GTTCGAGGCCAACCTAGTCTACAGAGTGAGTTCCAGGACAGCCAGGGATACACAGAGAAACCCTGTCTTG~~
~~AAAAACCAAGAAAAAATTAAAATAAAAAATAAATAAATAAATCTTTGGGCTGGAGCGAGCAGGGACTGAG~~
~~CGAGCGGGGCTAACCAGAGCAAGCAGGGTTGACTGGAGCGAGTAGAGGTCCTAAATACAATTCCCAACAA~~
~~CCACATGAAGGCTCAACAACTGTACAACTACAGTGTACTCATACACATAAAAATAAATAAATAAATCTTA~~
~~AAAAAAGAAAGAAAGAATTGTACACACTCACCTGCAAAGCCAGTAAAGGTCTCCAGCCTTCAGGTCCCTG~~
~~GAATGCTGGGCCTGACTAGGCTAGAAGCAGAGGTGACCTCCCTCCTCTCATCCCCATCCATCTCCCCTAC~~
~~AGTGATAACCCTTCAGTGAGCAGCCGGCCCTGGCATCCCTGCCTGTCGGGAGTGACCAGCCCAAGTCTAT~~
~~ACAGCAAGACCCTTCACTCCTGGATCCAGAGAGAACATAATGCTTTCAATCTGCTGCTAATAAAAATCCA~~
~~TTTGGCTTTATGTCTCTGTCTGTCTGTCTGTCTGTCCTGAGCTCCTGAAGGAACGACAAAGCAGGCACAG~~

~~GCCTGCATTACCCTAGGATCTCCAGGGTGGCAAGACAGCCACAGGTGATTTCCCAGTAGTGAGGAGCAAG~~
~~CATGGGAGGGTGGGATGCTGACTCAGGCTTGCTGAGCTACAGCCAAACTCCATCCAGATTGTGGACCAA~~
~~GGTAGACCTCAGGACCAGGAAAGAAATGGGCATCGTCTGCATCTGAATCCTTCCAAGGTACGACCAGCCC~~
~~CAGCCCCAGACAGAAGAGAAGCTGAGTTTGCAAGGGGCTTGTCTTAATTGGGGTTTCCACTGCTGCAATG~~
~~AAACACCATGACCAAAAAAGCAAGTTGGGGAGGAAAAGATTTATTTGGTTAATACATTCATGTCATTCAT~~
~~CACTGAAGGAAGTCAGGACAGGAACTCACACAGGGCAGGAACCTGGAGGCCGGAGCTGATGCAGAGGCCA~~
~~TGGAAGATGCTACTGGCTTGCTCAGCCTGCTTTCTAATAGAACCCAAGACTGTGGTCTCTGCAGCCCAGG~~
~~GATGACACCATCCACAGTGGGCTCCTCCCACCCCAATCAATAACTAGTTAAAAAAAAAAAGTCTTACAGA~~
~~TGGATCTTTTTTCTTTTTTAAACTTATTTATTTACTATATGTAAGTACACTGTAGCTGTCTCCAGACACA~~
~~CCAGAAGAGGGCATCAGATCCCATTACAGATGGTTGTGAGCCACCATGTGCTTGCTGAGATTTGAACTCA~~
~~GAAGAGCAGTCAAAGCTCTTAACCACTGAGCCATCTCTCCAGCCCCTTACAGCTGGATCTTAAGGAGGCA~~
~~TTTCTTCAATTAAGGTTCCATCCACTCAGATAACTAGTTTTATGTAAAGTTGATGGAAGACTAGCCAGCA~~
~~TTGGGAGTCAAGGCCTTGAAGCCTTAGCTAACGTGGAAGCGTGGAGGAAGCAAGGCGTAGTAATCGAGTG~~
~~CACAGGGTAAGGTCAGCCATGCACCGACACAGCTCCCAGCCAGCATCGTATAGGCTCTCCAGCAAGGCAT~~
~~GTGTGCCCCACTCAGACAAGCAGCATAACGCACAGCTCCTGCCAATGCTGACAATCAGCCCAGATTCACA~~
~~TCAGACTCCAGCTTCAGATACCAGCTCCAAGTCTAGCGCCGCCCCACTTCCACCGTCCCTTTGTAACTAG~~
~~AAGTAGAGATGTTCTAAAGAGAGACTCATGACCCCTCCCTTTGGCTATCAGGCTACTGAGACATTCTCAC~~
~~TCTGGGAGAATCAAAGGGCGGAAGCTCTGTCCAGTCAGGAGAAATCTGTCTCTGGTGCTGGTTATTTGTT~~
~~TTTATTTTTGTTTTGTTTTTCTGTCAACTTGACCCAAGCCAAGGTCACCCAGGGAGAGGGAACCTCGTTT~~
~~GAGATTCCCTCAGATTGGCTTGTAGACATGTAGGACATTGTCTTGATTAGTCATTGGTGTGGGAGAACCC~~
~~AGGTCACTGTGGGTGGTGCCATCCCTGGGCAGGTAGCCCTGTAGGTATATTAGAAAATAAGTAAATAAAA~~
~~CTAGGCGAAACAAGTCAGGGTGAGCAAGCCAGTAAAGTTCCCGCATGGTCTCTGCTTCAGTTCCTGCTCT~~
~~GAATTCCTGCCCTTCAAAATGGACTACAACTGTAACTGTAATAAGCCCTTTCCTCACCACAAGTAGCTTT~~
~~TGATCATGGTCATTATCACAGCAATGCAAACTTCCCAAGAGGAGAGTTCACATATGCTGACAGCTTACCA~~
~~GACCACCTTACCTTTGGATAGATTGGAGAAGAGACAATGTCAGGTCAGTGATGGGCGGGACCACATCTTG~~
~~CCCAGGGCTGCTCAGCTGTGTATGCCTCTTGTCCCCTATGTAAGTCTAAACCTCACCCAGGACCTCAAAG~~
~~ATGGAGCTAGGGAATCAATGGGCCTCTTTATTTGTTCCTCAGCTTGTAACTACTAAATGATCGTTAATTG~~
~~GTGTGCTGAGGGCAGGTGGCTCAGCCTGGCTTGTTAGGACTGCCAGAGCCCAGGCCTTGACACTAATTAC~~
~~TCCAGAAATACCTTCAAGTTAAATAAGCCGTTAGAATAACTCATAAAAGCACTTCAAGCACTGAGCTTAT~~
~~AAAGGGTCATGAAGGACAGAGATCAAGAGTAGCCAGATTGTCTTACTCGATGACCTATGGCCATAAAGAG~~
~~ACACCATGGCCATGACAACTCTTATAAGATTTTAATTTAATTTAATTGGGGCTTACATTTAATTGGCTTA~~
~~TATTTTCAGAGGTTTGGTCCATCATCATCAAGATGGAAGCCTGATGACACATAGGCAGATGTGGTGCTGG~~
~~AAAAGGAGCTGCAAGTTCTACATCAGAGCCCACAGGCAGCATGAAGAGAGGGTGCCACTGGCCCTGGCTT~~
~~GGGCTTTTGAAATCCCAAAGGCCACCCCTAGTAACACATTTCTTCCAACAATGAGGCTATACCTCCCAAT~~
~~CCCTAATGGCCACACATTTAAATCTTTGAGCCTAAGGGGACCACTCTTATTCAAAACCCCACACAGATGA~~
~~AGAGAAGTAAGGAGGGGCCAAGGGGTGGAAGATGCAGGAATGTCCCTATGGACAGACACCCACCAGGCA~~
~~CACCAGAAATCTTCCTCATCATTCAAGTCCAGAGCTTTTAGGAAGTTTCATTACATAATCATGATCGATT~~
~~AAACTACTGACTGTCGGTGACTGAATTCAGCCACCATCCCCTCTCTCGTACCTGGAGGTCATGAGTTGAT~~
~~CTGGGAGGTCCAGCCCACACTAATCCTGTGCCTGGTCTTTGTGGTGACCAGCCCATCTGAACCTGCAGAA~~
~~GTTCCCATATGATCAGTCCCCATTTGAAGCTATGGAAACCACCCTTTCAAGTCTCTATGCTGGAAGATA~~
~~AACATCTTGTCACACAAAGAAGCACATGAAAAAAGCAAAAGGAAGTCAGCACGGGCAAGGCAATTTCTTT~~
~~TCTGCAAAAAGAAGCATGCATCAGAAGCCAAACCAGGCTAGAAAGCAGCACTCTTAGCCAAAATGGCCTC~~
~~CAGCTCTCATCTCTGATGTAAATGGTGACTGTGTTTCTTCCCGTTGGTGGAGCTCAACTTCATAACTGA~~
~~TGGGATTGACATTTGACGCAATTAGCTAATGGGTATAAAGAGGGAGGAGGGGACTTGGGAAATATGGGTC~~
~~CTTGTTAGGCTAGCTACCTGGGTAGAAAAACACAAAACAATATTTCTTACACCCATCTGAAGCTATGATT~~
~~TGGCTGCATCTCAAATGAACTGGATCAGACTAGGGTAGTGTTTTATAGCAATTTCCCCTGAGACAAAAAT~~
~~CCTCCATCTTGGAGAGAATCCTAGAAAGTCATGGGATTTTCTAAAAAGGGGTGCCAGCTAAAACTCAAAA~~
~~GCTCCATCCTGCAGAGAAGCTAGTTAAAACTCAGGAAGTAAACAACACAAAACTTTCAGGAAGTTTCTGA~~
~~AACTGATCACATGTGCTAGGCTCCTCCTTCCTCAAATGTATATAAGCAGTAAAAACTGCTGAAAGACACT~~
~~CTTGGGCCAGTAAGCCATATGGAAAAAAACTCAGACCAGCAAGCTACTTGTAAAGGACCCTCCCCAGCCC~~
~~ATGGAGCTGCCTACCTGCTGCACAGTATGCTCCAGGTTCCCAGTTTTCATGAGCCGTTGCTCACACTAGG~~
~~GTGGGCTTCGGTAGTTAAGTCGTCTCGAGTCACTTCTGCTCCTGTAAGTAACCCTCTGTGGTGGTGTGAA~~

~~TGAGAACGGCCCCATAGGTTCCTGTGCTTGGATGCATGGTCATCAGCCGGTGGAACTCTTTGGGAAGGAC~~
~~TAGGGGCTGTGACCTTTTGGAGATGTGTCACCAAGGTTTCAAAAGTCCATGCTATTCCCAGTGTAGGTTC~~
~~TTTCTCTCTCTGCCTCCTACTGTGGATCAAGATGCAAACCTTCAGCTACTGCTTCAGTGCCAAGCCTGCT~~
~~TACTTCCATGCTCCTTGTCATGATGGTCATGGACTTTAACCCTCTGAAACAGTAAACTCTCAATAAACTC~~
~~TTTCTGTATGTTGTCTTGATCATAGTGGCTTATCACCGCAATAGGAAAGTAACTAAGACACCTTCACTCA~~
~~TACTGCTATGAGTAACCCAAAGAACACTCATTGTTTCATCAAGCTGGACTTTGGCAGTCTCTTACTGTGA~~
~~TCCATCATTGGTTCCCTATCTGGGCTGAGTAGATATTTGTTCATGTCTCTCCTTTCTTGCCTCCTACCAG~~
~~TCATAATAAAGGAAGGTCACATGGCTTTGCCATCTTAAGGGCCAAATACTGGGAAGATTAATTTTAATT~~
~~GTTAATTTGACTGAATTTAGAATCAGCATGGAGGAGCTGGAGAGATGGCTCAGTGGTTAAGAGCACTGGT~~
~~TGTTCTTCCAGGAGTCTGAGTTCAATTCCCAGCAACCACATGGTGGCTCACAACCATCTATAATGGAATC~~
~~TGATGCCCTCTTCTGATGTGTCTGAAGACCGTGACAGTATACTCACATATATAAAATAAATAAATCATTA~~
~~GAAAAAAAAAGATACCAGGCAGTGGTGGCCTTTAATCCCAGCACTTGGGAGGCAAAGGTGTGCTGATTAA~~
~~GATGTTTCTAGACTGCTTGCTGACAGGAGCGTGATAGAGCTGTCTCATGAGAGGCTCTGCCAGTGCCTGA~~
~~CAAATACACAAGTGGATGCTCACAGCCTTCCATTGGACTGAGCACAGGGTCCCCAATGAAGGAGCTAGAG~~
~~AAAGGATCCAAGGAGCTGAAGGGGTTTGCAGCCCCATAGGAGGAACAATATGAACCAACCAGTACCCCCA~~
~~GAGCTCCCTGGGACTAAACCACCAACCAAAGAGTACACATGGTGGGACTCATGGCTCCAGCTGCATATGT~~
~~GTCAGAGGATGGCCTAGTCAGTCATCAATGGGAGGAGAGGCCCCTTAGTCCTGTGAGGACTCTATGCCCCA~~
~~GTATAGGGAAATGCCAGGGCCAGGAAGTGGGTATGGGTAGGTTTGTGAGCAGGGGGAAGGGGAATGGGAT~~
~~AGGGGGTTTGGGGAGGCAAAATCAGGAAAGGGGATATTAACATTTGAAATGTAAATAAAGGAAACATCCA~~
~~ATAAAAAATAATTGTTCTGGGCTGGTGAGATGGCTCAGCAGTTAAGAGAACTGACTGCTCTTCCAGAGGT~~
~~CCTGAGTTCAATTCCCAGCAACCACATGGTGGCTTACAACCATCTGTAATGAGATCTGATGCCCTCTTCT~~
~~GTTTGAAGACAGCTACAGTGTACTCACATACATAAAATAAGTAAATAATTTTTAAAAGGAAGGAAGGAAA~~
~~GAAAGAAGGAAGAGAAATAATTTCACTTCCATTTGGGACTCAGAAAAAGTATAGATATCTGTCAAATAGA~~
~~AAAACACAAATTTGGTAAAAGAACAAAAAAAAAAATTGTTCTTCTCATACACTGAAAAAAAAAAAAGATGTT~~
~~TCTAGATGACTTTAACAAGGAGAAAAGACCCATGTGAATGGGAGGGAAGGGGTGGAGAACACCATTCTGG~~
~~GGACTGGAATACCAAAATAAATAAAGAGAAGAAAGTCAAAGCCAGGCATGGTGGCACACATATTATTATT~~
~~ATTATTATTATTATTATTAATTTAAAGTATGACTGCTCTGATGCATATACACCCTCAGGCCAGAAGAGGG~~
~~CACCAGTTCCCTTATAGATGGTTGTGAGCCACCATGTAGTTGCAGGAATTGAACTTAGGACCTGTGGAAG~~
~~AGCAGCCAGTTCTCTTAACCTCTGAGCCATCTCACACACATCTTTAATTACATCATGCAAAAGGCAGAGG~~
~~CAGGAGGATCTCTGAGTCTGAGGCCAGCCAGTGAGCTCCAGGGCAGCCAGGACTGCTCTAAAGACTGTCT~~
~~CTAACCCTCTCCCCCCACCCTGCAAAAAAAAAAATAAAAAATATAGCTGACCATCAACATTCATTTCTCTC~~
~~TGCTTCCTGACTATGCCTTTTGGGTCTGAGGTGAGGAGGTGCAGTAGCTCTGCTGGCTACTGTGTGCTCA~~
~~GGTGGCTCAGCAGGTCACCTGTGCATGGAGCTCAAGGCCAGTTCAGTTTCAGCATCCACACCATTCACGT~~
~~TAGGACCAGTGGTCCTTCTTCCATTCCCTCAAGTCACTGCAGGACTAGGCACCTCCTCCCCACTGAGGCC~~
~~AGATGAGGCAACCCAGTTAGGGGAACAATATCCACAGGCAGGCAACAGAGTCAGGGTAAGCCTTAAGAAG~~
~~CATTCTTGGGGCTGGGGAGATGGCTCAGTGGTTAAGAGCACCGACTGCTCTTCCAGAGGTCCTGAGTTCA~~
~~ATTCCCAGCAACCACATGGTGGCTCTCAACCATCTGTAATGGAGTTTGATGCCCTCTTCTGGTGTGTCTG~~
~~AAGACAACTACAGTGTACTCATATACATTAAATAAATAAATCTTAAAAAAAAAAAGCATTCTTTACAGCA~~
~~CGTTGGTCAATACAATGCCCATGTCCAGATTACAACCTGATCCTGGTCACAGAACTCAGCAGCATCTAAG~~
~~CATATCTTGCACAAGACCAACCCTAGCAATGGCCTCTGCTCCAGTTTCTGCCTTGAGCTCCTGTCCTTTC~~
~~TTTGATGATGATGTGTGATGTGAGTCAAGTAAACCACAAGTTGCTTTGGCTATGGTTTTTATCACAGTGA~~
~~TAGCAACCTAACAAAGACATTGGCCAAAGTCCATCTTAAATCCCCTAACACTTTGGGGTTCCTTGCTCCT~~
~~TTCCTAAAGCTCTACTCCCCAGCACAAGGTTGCCTGACAAACATGGTGGCTGACCCTATCTGAGCTGCAT~~
~~GGCTCTAGCTCCCGGCCACATGGATGCCTGCCACCCGGTGGCTAACCTGTGAACTGCCCAGCTCAAGCTG~~
~~CAGGGCTATTCTCCAGTCCCCAGGCAGCTTTGAGGTTTATGGCTGACCGGCCCCGGGGTTTTCTTTTTGA~~
~~ACTCTAACTGTCCTAGGCTTCCATTTGTGTGTATGTCCTAAATTATTTTATTAATAACCCTAAGGAAGAA~~
~~CCCTGATTCCCTAGGCGCCATAGAATGGCTCTCCTGGGGTTCCCCAAGATCCCCAATAACACATCTTTCC~~
~~TACTTAATTATTTAATTGGCAGAGAAAATGAACCCATTCCCATACTTCTAGCTGCCATAGTCCTGGGGCT~~
~~ACAAGGCCTCTGGCACCTGGTCTAGCCTGGGGTTGGTTCCAGGAACTGTGGCGAGGTATAGGCATGAGAT~~
~~GAGGGTGGCTCACAGCTTTAAGGAGCCTTGAGTGGAGGTGCTGTCTTCAGAGGCTCCAGCCTTTCACTGT~~
~~TACAGAATATCTGATCACACTGTGAACCCTAAGACTGTGTTACTTACTGGAAAACCTGTTTGTAGTTGTG~~
~~GCAGCTCTTAGCACACAGCTCTAATCCCAAACAATGACAGTAAAGTAAGTTTGTAGAAGGAAGCACCCAT~~

~~GTTTGAAAGTGATGTCTACCTAATTGAGTGGCAGGTGAAGTGAAGAATCAGAGAAGGATCTGACAGAATA~~
~~TGATCTGCCCAACTCTCACAAGAGGAAAGGGAAGCTACTTGAGGGGCAGCGCAGAGAAAAGGAAGGAAGC~~
~~GGTTTTACTGCGACAGTTGTACAGAGGCAGATTGCCGAGTGAATAAATTAGACACAAGTGAAGACAGGAG~~
~~CCAGAGAATGAGGACCCAGAAGATTACAACAGATTGCCAAAGTTAGTAGGAGTTCAAGCAAAACAGTCAA~~
~~GAGGCCAAGAAAGAAGCCAGCTTGAATCAGTCAGTGTGGAGAGTGTGGAGAGTGTGGAGAGGAGTTTTGA~~
~~GCCAGAACAGCTGAGTTGACCAGCTCAAAAGAAGCTAGAAAGGGTGAGCTTATTCAGCAACAAGTCTCAG~~
~~AGGCTGAAAACATTCTAGGCCTAGATAAGATTCTACAGAGGCTAGAAGCTTCCAGGACTAGGCCTATTTA~~
~~GCAAACAGAGGCAGTAGGCCTCCAAGACAACAGTCCCAGCACTAAGAATTTTATACATTCTCAGCTGGCT~~
~~GTGGTGGTATGTCCACCACTGCAACCCTTCATTTCCTTTCTGGTCCATTTTATCTTTGTTTTGTTTGTTT~~
~~GTTTTTCCTTTTTGTCATTTTTGCTTTTGGTTTCCTACTAAGAAACTACTGTTTAATCCAAGATAGTAAA~~
~~AATGTGTTTCCCCTTAGGAGTTTTATATCTATATTATCTCTGACCTTTTTGAGCTAAATTTTGAATTCAG~~
~~CATGAGACTCTTAAACAAGAACCACCAAGGTAAAGCTAGTGTGGCAATATAATGCTAGCTACTGTAAGGC~~
~~TAAAGCAAGAGAAGCTTCCCTGGATAATTCAGCAATCTTTTATTAACAAGAACCAGCGTTTACGCTTGAT~~
~~TTTAGCCACAAAGGAGAGAAGTAATACACTCTTACAAACATAAAGATTTTTCAAGGAAAATTTTAAGTTA~~
~~AATTTTCCTTTCTACAAAATTAGGGCCAGCAGGATAACTTAGTGGGGACCTGGGGGTGGGGGTGGGGCAT~~
~~GTCTTTTGTCCTGCACTCCAGAGGCAGAAGGAGGGGGATCTCTGTGAGTTCAAGACCAGCCTGGTCTACA~~
~~CAGCGAGTTCCAAAACAGCCAAGGCTACAAGGAGAAACGCTGTCTAGGAAAACAGACAGTTAGTTTAGGG~~
~~GCCACTTGACTGTGAGTTCAATTCCCAGATCCCACATGATTGGAAGAAACAAATTTCCCCAAGTTTTCCT~~
~~GTGATCTCTTCATGCACACATACACCTTTGTCCCAGAGATGCCGTCTGTGGTTTCCTAGCACTGGATTA~~
~~CTGGGAAAACTCACCTCCCAACCATCCTACCCAGCATTTTGTACTATCTCCTCAGTCCTGCCGTGCGCTA~~
~~AGGCAGGGCCTTCTCCAGGTCGTCCCGGCTGTCCTAAACTTGGTATGTAGACCAAGCTAGCCACGACCTC~~
~~AAAGATCCTTTTTCCTGTCTCCTGGCTGGGGTTTTAAGGTGTGTGCCACCACGCCCGGCTATTCTGCTTC~~
~~TTGATGCAGGGCCTCCACAGTAGTACAGGCTAGCCCTCGGCCTCCTGCCTCCATTACTGAGATTACAGCA~~
~~ATTTATCGGTGTGCCGTTGTATACGGTATGTCCTTTACTTGTTTAATCTAGACAAACTGCTTGCATTTCC~~
~~GAAAAGACCTTCAGCTGGAGCTACAACTCGCTTCTCTGTAATTACCCGACACCTGGCAATGCCTTCCCGC~~
~~CCCGAACCCCAAAGTCAACACGGCGGTCCCAACTCACGCCTCTCCTACAGGACCTGTGAAAGGCCCAAGG~~
~~GAGTGGGGCAGAACGGGCTGACATCAGAGTCGCTACCCAAACTCCGCACAAGTAGGAGACTCTGGCCCCGA~~
~~CGTGCCCTGCCCGTGTAGAATTCACCCTCTCCTTCCCGTCTACGTTTCCCACGACCAGGATCTCACTACAC~~
~~TTCCCAGTACCGGAAGCCTGAGCGCAACAAAGATGGCGACTGCGGAAGGGGCGGAGGCGTGGCCTCCGT~~
~~CCGGGAGCACTGGGCCACCTAGCTTCCGGCGGCGCGCCAGTGAC~~**~~GTC~~**~~AGTGAGCGCGCC~~AGGCCGCGTGG
AGGCCGTAGGCTGATGGAGGTCAGTGAGGGTGATCGCCGCCGCAGTCTGGCGTCCTGGAGAGACCGGGTT
TCTGAACGTGGGTATCCGAGACGGGAAGCGTGGCTGGGAAGGGCAGCAGTTAGTAAGGACCGGCGGGGCT
GTTGTGGGGGGAGCCAGGAAAGGGACTCTCGACACCGTGCCCCAAGGGCGCTGTTCGGTTGCAAATTCGG
TTCC

E2/3-Tbrg4 (Δ17,874)

TCAGGTTTTTAGGGAAAATTCCAGCACAGCTGACCCCATAGGACAAAAAGTGCAGTGTGTGTACTACCAA
AACCCTGGTGTCCTTTTCCCACCCTAGCCGAGCAGAGTTGATGGGGCAGGAAAGAGCCTACGCATACTGG
AAGCACACAGGCTCAAGACTGGCAGGCCAAAGAACCAGAACACCCCAGGGCATAAGAACCCCATGCCCCT
TGCCGCTGGGCCTGGTGAATTCAGAGTAATGTCTTCA**TTCCTAGAA**CCTTCTGGCTTCCCGACCTGGCCT
GCAGGCTCTGAGGAGATGTGTGCACCTCATGAACTCCTTGTTCAGCCAGGGCCTCTCTGTCCTCCCTACA
CTTCCCCACCACACAGGAACACATGTCCTCAACTGACCAGTGTCACCCTGGGCCTCAAGGCTAGGTTCCC
TTGAGTACTGGGACACAAGAACAGACTTCTGCTCTCCCCTCTGTCCACACAGAGTGCAGAGAGGAAGGGT
TCTGGTTCATGTCCCACAGTTGCCCCCTAAAACCGATGTGATATAGCCCTCACTGGCCTAGAGCTCACTA
CATAAACCAGGCTGGCCTGGAACCCACAGATCTGTCTGCCTTTGACTCCTACTGCTGGGATCAAAGGTGT
GAGCCACCATGCAACGCTCTGAAACTGATTCTTTAGAAGCTAAGAAAATGCTTCAAAATGGCAGTAGCCT
TGGGCTGGAGATGGGTTAGTGGTTAAGAGAACTGACTGCTCTTCCAGAGAGAGAGAGAGAGAGAGAGAGA
GAGAGAGAGAGAGAATGAATGAATGAATCCAGAGTGCTTTCTTCATACATGCTAGGCAAAAGCTCTACTA
CTGACCTGCAGCCCTAGCCAGATACATATATGCATATACACATAGATACACACACACATGTGTATTATTC
TCTCCATGTAGTCCTTGGCTGTTCTGGAACTTACTCTGCAGATCAGGCTGGCCTCAAACTCTGCCTTCCA

AGGGATGGCTTGTGCCACCATGCCTGACCAGAGATTATCTTTCCAAGCTATTTACCTTCCCTGCTTGAAA
GCGCTCTCACTCTTTCTCATTATGTTTTTAATCCCCAGTCTGGTCCAGTGTGAAAGATGCTGTTGCTCCC
TCGATTTGCAGGGAGCTTGGGGCATGGGAGCAAATGACTTACCTGAAGTAGAGTGGAGACAAGGGCGAAT
CCCCAGGGCAGCTCAGCCTAGGGGCCTTGGGACTGAAAGGACAGGAAAAGAGGAGGGACTGGGGAGCTGT
GGTGCAGGGTAGCCTGAGGGAGGGATTGTAGAGGCCAGAGGAAAAGACTAGTCTAACCCAGTGCCTAGCC
CATGAGCAGGAGCAGCACTGGGCCGTTGTTAACACACAGGAGGATTTTATCCGTAGGGATGACCCTTGGT
CTCCCAGCCCCATACAAAAGTATTTAGGGACAGAACCTCAAGGGTTAATTGTGGCAGCACAGGCTCTGGC
TCAGGACCGAGGTTTTCAACTGTGAATCCAGGCACTGGCACTGAAAGGTGGGTGGTTGTTTGTTTGTTTG
TTTGTTTTGGGTTTTTTTGTTGGTTTGTTTGTTTACTATTTCTCCTTTATATGTTAAAACCACGACTTTA
AAAAGCAATGGGGGCAGGGAGGGAATGGTTCGGTATTAAAGAGCACTGGCTGCTCTTTCAGCTCCCTGCT
CCCACATGGCAGCTCACAACCATCTGTAACTCCAGTTCCAGAAGATCTGATGCCCTCTTTTGGCATCACA
TATACACAGACATATACACAGGCAAAACACACATACACATAAAGTAAAAATAGTACATTAGTATTAGTGG
GCTGGTTAGAAGCTGAGCAGATAAAAGTATTTGTCATACAAGCCTCACTTGTCTTACTTTGATCAATGGA
GCCCACAGGAGAAGGAGAGAACCAGCCCCTGAAAGTTGTCCTCTGACCTCCACACACACTCACACACACA
TACACACGTACTCACATACACACATACACACGTACTCACACACACGCACATACACACGTACTCACACACA
CACAAACACACGTACTCACACACACACACACATACACACGTACTCACACACACACTCAAACGCACACACC
ATAGCACACATGCTGTACATTCACTCACCTCTCTGTACACACACACTATAGACATACTAATAATAAATAA
ACAATGAAGTAGTGGTGGCGCCTGCATCTTCAACTATCAGTCTATCCGAGTGGACACGATGAGTGTGCTT
AATGGCTGGTGTTGAGGAGATGGACTCCTGGCCATCTGTGCCAGCGGGAGTCCAAAATTGGGACAGTCTC
CTGGGAGAATCCTAGACAGGACTGGCCACATGGGCAAAACATGAAACCTGTTTCTCAACAATGTTGCTTC
TGTATTTGAACCCTGCAGAGCCCTTTTGGCCCCAGTATACATTTGTAACAGTAACAAATCAGGGCCTGGG
GGTGGGGAGGAGGGGAGAGGGGTCAGAGCAAAGAGCTTGCTATACTTGCAGAGGACCCAGCATCGATTCC
CAACCTCTGCATGGTGGATCACAAACACCCAGAACTCCAGTCCTAGAGAGTCTGATGCCCTCTTCTGGCC
TCCATGGGCACCAGGCATACATGACATGGTACACAAAAATACAGACAGACAAAACACTCAAGCACTACAT
GTAAAATATAATAAAATAAAATAAGGAATCAGGAACAATCTAAATTTCCTGGATGGGCCAGGAAATACAT
GGTAGCATCCATAATGCTATGCAAATCAACCAGCCATCCAACAGTGTATCTGTGTATCAATAGAGGGATG
AGTTTCCACGATACCTCTAAGTAGAGGGGAAGAAAAGGAGAAGTTATCTTCCCAAAAGCTGAACTATGTA
TCCATGTTTGAACTTGTCTTGATTATCTCCAAAAATGGAAAACAAAAACAAAAACAAAAACCTCGAGGAA
CGAGGTTGCTGGAGCTGAAGCCTGAGGCAAGCCACTTCCCTTCCAGGAGGTTCAACTCTCTCTTCCTGTG
GCTCTGAAAGAGTATAAGAGGACCTGGAGAGACCTGAGCTTACATATATACACATATACACACCTGGAGA
GACCTGAGCTTACATATATACACATATACACACCTGGAGAGACCTGAGCTTACATATATACACATGTGCA
CACACACACACTTGTACTTACACTCATATGCATACATACATACACACAGTCACATACACAAACACTTGTA
TACACTCATATATACCTATACACACTCACATACATGCACATACATATACACACGCTCACACACACAAACA
TAAGCTGAAGCAAGATGGCGGAGTTGAGCCTGGCCTTCTGCCGGCCACAGGGTGAGCTCAACCTGTCTGC
TGGTTCCTGTCCTCTCTGAAGCTAGAATTCTTTCACTGCTAAAACAGGGCGGGAGGAGTCCAGAGCCCTG
CCACTGGGTGCAGAACATGAAGACCCCTTGAGTGGAAAGGGGTTATACAGCTGGACAGTGGTGGCGCACA
CCTTTAATCCCAGCACTCGGGAGGCAGAGGCAGACGGATCTCTGAGTTCGAGGCCAGCCTGGTCTACAGA
ATGAGTTCCGGGACAGCCAGGGCTACACAGAGAAACTCTTGTCTCGAAAAACCAAAAAAAAACAAAAAAG
GAAAGGGGTTACACAACAGAGACTCAGGTCACAGCTACCCATCACACACAGGATACACATCCAAAGGTGT
TCACAGGCAGATGAGGAACGAGGAGAAGGGGCTCAAGCAAGGGCCTAAAGTTTCTTTTTTTTTTTTTTTT
CTTTTTTTTTTTCCCTGTGGCCTAGAGTTTCAAGAGGCTGAGGACCTAGGCATGAACCAAGAGGGGCCAA
ACCACTTCAAGAAGCAGGGGGTAGCAGCAGAATCTCACTATCAGCCTTGAGCACAGCTGGGAAGGAGATC
CATGGAAACAACCAAGAAAGAGCTGAAAGGGGCTGGAGAGATGGCTCAGCAGTTAAGAGCACTGAGTGCT
CTTCCGAAGGTCCTGAGTTCAAATCCCAGCAACCATATGGTGGGTCACAACCATCTGTAATGAGATCTGA
TGCCCTCTTCTGGTGTGTCTGAAGACAGCTACAGTGTACTTATGTATAATAAATAAATAAATCTTTAGAA
AGGGAGGGAGGGAGGGAGGGGGGGAGAGAGAGAGAGAGAGAGAGAGAGAGAGAGAGAGAGAGAGAGAGAG
AGAGAGAGAGAGAGAGAGAGAGCTGGAAGAGGGAGATCTGGGAAGTCTGCTGGCTTTATATGCTGACCAT
ATATAGTCACCTGTGTTTACAACTGTGCTCATCACTTTGAAATCTCAGTGGTTTCTCCTTTGAGCCTGTG
TCTGTAAGTACACCAGGACAGTGGTACATAGGCAAGAATAACAGCCAGTGGGCATAGGACACAGAGTGCA
TGGGCCCCAGCAAGATGCAGAGAGAACAGAGCTCTGGCTCCTAAGACACAGGGCC**TTCTGGGAA**ACTCAA
GCAGCCAAGCAACCCTAGCCAGCCCTTTCCTGGTGGCCCTCCTTCTGTTCCAGCAAAGGCGGAAATGGGA
ACAGGGGTGGAAGCAGAGCATTGGCAGAGCATAGGTATGACTTAGTCTTGACTAACACAAGCATGGCAGT

AGCCTGACAGTGGCCTAAATGTGGGGATGACTGCCTTAGATGAGGATGACTGCCTTAGATGGGGATGACT
GCCTTAGATGGGGATGACTGCCTTAGATGGGGATGACTGCCTTAGATGGAACAACAAACATCTATGGGCA
TGCTGTGGAACACTGGCCCACACACGGAACTGAAGCACTGGCAATTTCCACAGGGCAGTTAAACCTAAAA
GCATGCTCACACTCAACAGGCTGCCGGAACTCATGAGACACCTGGAATAGACGAATGTAGAAACAGAGCA
GAGAGGTGGTTGCCAAGGTCTGGGGGCTCAGAGGACAAGCAAGAGGCGCGGCTTTCCTTTGGGGCTGGCA
TGAAAGGAAATATCGAGGTTACAGCCTGAGAGGGCTTCCCCTGACACTTCGTATTCAAAGAGGCCATGGG
CCACCAGTGAAGACAAAGGAGTATGGCCTGCACCACAGGCTGGCGCTGACAGTCAGTAAGCACACAGTCA
CTCTGGGTCATCCCATCCCCTTCCTTGCAAGAGAAATCAAGGAAATGTCCCGAGAACAATGGGCACAGT
GCCCAGCAGGACATCTCTTCCTGCCCATGACACCCTTGGCACAGTATGGCCC**TTCTGGGAA**GTGCCCTT
CCAATGTGCTCTGCACAGGCAGCTCCTTTTCAATGTATGCCCGACACTCTCTACATGGAGCAAGCGCCTC
CACACTCTTAGAAGAATTTTTAGAAAACTCCAGAAAAGCACCAGGAGAAGTCACCCTCAGATGTAGCCCG
GACTCGAGCCTTGCTCAAAACCTCCTGTCTTGTTTTCTATGTGACTGTACAAATTTGGAGCTCAGAATTG
CCTTTGTCTGTGATGGGTTCCAACCCAACCACTCAAAGTGACACTTGTCACATTTGTCACTGATCCTATT
TCTTCTTTTTCTGCTCCTTCATTTTCTCCGCTTTCATAATAAACAAGTATTACTTTTTAAGTGGGGAAA
AAATGACCACCCTTACAAAGGACTTTTTAAAAATGGCCTCCATTGTGGCCCTTGTTCCTGGCAGCCTGGG
CCTGCTCTCTCTGTGTGGCCAAGAAGGAAGTGTTGTAGCCCATCTAGAGCTGTGCCAGCCTCTTCCCCCA
CCCCACCCCCAAAGTCTTCCTCCTGTGGGTCCTTTAAATGCATCCCAGACACTCAGACAGCC**ATC**AGTCA
CTTGCCTGACACCGGTACCATGCGTTGCCTCATCAGCCTTGTTCTTGGCCTGCTGGCCCTGGAGGTGGCC
CTCGCTCAGAACCTAGAGGAACAAGTCTTCAACTCAGGTAAGCCCCAGTTTCTCACCTTACTACCGGGTG
TGGCTTGGAGAACCACACTCACACATACTGATCCCAGCTGTCTATGCTGAGGAGGCAAGGAACAAAAGAA
CACCCCTAGAGAAGAATCCAAGGGTGGCAGGAAGTAGGGGCCTGTCCCACACAAGCTGTACAGCCAGGAA
CCTGGGTGGAAATGTTCAGGCTAAGAGACAGGTTGATGAGAGTTACCCTCAGGCCCTGGTCACTTCTGGT
CAGATTCTTAATGTTCTACCTATAATGATGAGCCTCTGACCTAATGCTCTGCCCAGGTCCATGAGCCCAT
GACCTAATGCTCTGCCCAGGACCATGAGCCCATGGCCTAATGCTCTGCCCAGGTCCATGAGCCCATGGCC
TAATGCTCTGCCAGGTCCATGAGCCCATGGCCTAATGCTCTGCCAGGTCCATGAGCCTCTGACCTAATGC
TCTGCCCAGGTCCATGAGCCTCTGACCTAATGCTCTGCCCAGGACCATGAGCCCATGGCCTAATGCCCTG
CCCAGGACCATGAGCCTGTGGCCTAATGCTCTGCCCAGGTCCATGAGCCCATGGCCTAATGCTCTGCCCA
GGTCCATGAGCCTGTGACCTAATGCTCTGCCCAGGACCATGAGCCCATGGCCTAATGCTCTGCCCAGGTC
CATGAGTCCATGGCCTAATGCTCTGCCCAGGTCCATGAGCCTGTGACCTAATGCTCTTCCCAGTTCCATG
AGCCCATGGCCTAATGCCCTGCCCAGGACCATGAGCCTGTGGCCTAATGCTCTGTCCAGGTCCATGAGCC
CATGGCCTAATGTTCTGCCCAGGTCCATGAGCCTCTGACCTAATGCTCTGCCCAGGTCCATGAGCCTCTG
ACCTAATGCTCTGCCCAGGTCCATGAGCCTCTGACCTAATGCTCTGCCAGGTCCATGAGCCTGTGGCCTA
ATGCTCTGCCCAGGTCCATGAGCCCATGATATGCTTCCTAACTCTCACAGCTACTTTCTCATTCTGGCAG
GTTTTCTGGTTTTCTTAGTTTGGCTTGGTTTGGTTTGCTTTGCTTTGGTTTGGTTTGGTTTGGTTTGTTC
ACCTTTAAGGCTCCTCAAAGGCTTGACTTTTTGTAGCTTCTATCCATGTCTCCATGCCTTCTTCTGAGAA
GCCTTAGAAGTGAGGTTGATGTCTGGCCCCCATTTACCTCCACGTCTTTTGTGCAGTTCAGTCCATGTT
CCCAAAAGCCAGCCCCATTGAGGGCACAGAGTGTATCATCTGCCAAACCAACGAGGAGTGTGCCCAGAAT
GCCATGTGCTGTCCCGGTTCCTGTGGTAGGACCCGCAAAACTCCTGTCAACAGTAAGTCCCCGCCCAATC
CCGGTGGTGGGAGGAGCCAGCAGGGTCAAGAGTCTGCCTGGAGAGGACACATAGTCCTTTACCCACACA
CCAACTCTGTGAACCCCACTTGCCTGTCAGACCCTCCCTGCAGCCTGCTGTCCATCCTAGCCCCTGGGGT
CTTGAGCTTCCAAGGGCGCTGCCTGACCATAAGACAGACTTCAACAAATGCCCCATTCATGAAATAGGGC
TTTTCAGTCATGGGGTCAGTAAAGTGCACTCAGCAAATATGGGGCATGAAAGGGCTTTGGGTGGTAAGAG
TGACCATCTCTCCCCAGTTGGTGTTCCGAAAGCTGGCTTCTGCCCTTGGAATCTACTCCAAATGATCAGT
AGTACCGGGCCGTGCCCAATGAAGATAGAATGCTCCAGCGACCGTGAGTGTTCTGGCAACATGAAATGCT
GCAACGTCGACTGTGTCATGACATGTACACCCCCAGTGCCAGGTATGCAGTCTCAAAACCCTGGACCCCC
ACTTCTCCCTAGATCTCCTCTGGCCCCCACCTTACCCTGCATCAGATCCAAGTGCAGATACAATGTGAGA
CCAGCCCAGGACTAGGTCCCAGGGTAGGAGGAGAATGTCCTAGCCAATCCTGCAAGCCTGTGGGACTTT
CTGTGGCCACTCACTGTAGAAGTGAATGTCAATGAACAATGGACCTGAGAGAACAGACCATTCTAGCACT
CAAAACTAGTCCTGGTGAGTCTATGGCCACATGGTGCCTTAGGCACTGAACCTGAGCAAACAGTAAAGT
GCTCAAAGTTAAGAACATGAGCTCACATCCGGCTGCCCATGTCCACCTGGGCGCCCCCTGCAGGCGGGCG
TGTCTCACCGACCTGTCTATGGGTGATGGGGGCACTGTAAAGAATTGGGCTGGAGAGATGGCTTAGCAGG
TAAGAGCACGGACTGCTCTTCCAAAGGTCCTGAGTTCAAATTCCAGCAACCACATGGTGGCTCACAACCA

~~TATGTAATGAGATCTGACGACCTCTTCTGGTGCGTCTGAAGACAGCTACAGTGTACTTAGGTATAAAAAT~~
~~AAATCGCCGGGCAGTGGTGGCGCACACCTTGATCCCAGTACTTGGGAGGCAGAGGCAGGCAGATTTCTGA~~
~~GTTCGAGGCCAACCTAGTCTACAGAGTGAGTTCCAGGACAGCCAGGGATACACAGAGAAACCCTGTCTTG~~
~~AAAAACCAAGAAAAAATTAAAATAAAAAATAAATAAATAAATCTTTGGGCTGGAGCGAGCAGGGACTGAG~~
~~CGAGCGGGGCTAACCAGAGCAAGCAGGGTTGACTGGAGCGAGTAGAGGTCCTAAATACAATTCCCAACAA~~
~~CCACATGAAGGCTCAACAACTGTACAACTACAGTGTACTCATACACATAAAAATAAATAAATAAATCTTA~~
~~AAAAAAGAAAGAAAGAATTGTACACACTCACCTGCAAAGCCAGTAAAGGTCTCCAGCCTTCAGGTCCCTG~~
~~GAATGCTGGGCCTGACTAGGCTAGAAGCAGAGGTGACCTCCCTCCTCTCATCCCCATCCATCTCCCCTAC~~
~~AGTGATAACCCTTCAGTGAGCAGCCGGCCCTGGCATCCCTGCCTGTCGGGAGTGACCAGCCCAAGTCTAT~~
~~ACAGCAAGACCCTTCACTCCTGGATCCAGAGAGAACATAATGCTTTCAATCTGCTGCTAATAAAAATCCA~~
~~TTTGGCTTTATGTCTCTGTCTGTCTGTCTGTCTGTCCTGAGCTCCTGAAGGAACGACAAAGCAGGCACAG~~
~~GCCTGCATTACCCTAGGATCTCCAGGGTGGCAAGACAGCCACAGGTGATTTCCCAGTAGTGAGGAGCAAG~~
~~CATGGGAGGGTGGGATGCTGACTCAGGCTTGCTGAGCTACAGCCAAACTCCATCCAGATTGTGGGACCAA~~
~~GGTAGACCTCAGGACCAGGAAAGAAATGGGCATCGTCTGCATCTGAATCCTTCCAAGGTACGACCAGCCC~~
~~CAGCCCCAGACAGAAGAGAAGCTGAGTTTGCAAGGGGCTTGTCTTAATTGGGGTTTCCACTGCTGCAATG~~
~~AAACACCATGACCAAAAAAGCAAGTTGGGGAGGAAAAGATTTATTTGGTTAATACATTCATGTCATTCAT~~
~~CACTGAAGGAAGTCAGGACAGGAACTCACACAGGGCAGGAACCTGGAGGCCGGAGCTGATGCAGAGGCCA~~
~~TGGAAGATGCTACTGGCTTGCTCAGCCTGCTTTCTAATAGAACCCAAGACTGTGGTCTCTGCAGCCCAGG~~
~~GATGACACCATCCACAGTGGGCTCCTCCCACCCCAATCAATAACTAGTTAAAAAAAAAAAGTCTTACAGA~~
~~TGGATCTTTTTTCTTTTTTAAACTTATTTATTTACTATATGTAAGTACACTGTAGCTGTCTCCAGACACA~~
~~CCAGAAGAGGGCATCAGATCCCATTACAGATGGTTGTGAGCCACCATGTGCTTGCTGAGATTTGAACTCA~~
~~GAAGAGCAGTCAAAGCTCTTAACCACTGAGCCATCTCTCCAGCCCCTTACAGCTGGATCTTAAGGAGGCA~~
~~TTTCTTCAATTAAGGTTCCATCCACTCAGATAACTAGTTTTATGTAAAGTTGATGGAAGACTAGCCAGCA~~
~~TTGGGAGTCAAGGCCTTGAAGCCTTAGCTAACGTGGAAGCGTGGAGGAAGCAAGGCGTAGTAATCGAGTG~~
~~CACAGGGTAAGGTCAGCCATGCACCGACACAGCTCCCAGCCAGCATCGTATAGGCTCTCCAGCAAGGCAT~~
~~GTGTGCCCCACTCAGACAAGCAGCATAACGCACAGCTCCTGCCAATGCTGACAATCAGCCCAGATTCACA~~
~~TCAGACTCCAGCTTCAGATACCAGCTCCAAGTCTAGCGCCGCCCCACTTCCACCGTCCCTTTGTAACTAG~~
~~AAGTAGAGATGTTCTAAAGAGAGACTCATGACCCCTCCCTTTGGCTATCAGGCTACTGAGACATTCTCAC~~
~~TCTGGGAGAATCAAAGGGCGGAAGCTCTGTCCAGTCAGGAGAAATCTGTCTCTGGTGCTGGTTATTTGTT~~
~~TTTATTTTTGTTTTGTTTTTCTGTCAACTTGACCCAAGCCAAGGTCACCCAGGGAGAGGGAACCTCGTTT~~
~~GAGATTCCCTCAGATTGGCTTGTAGACATGTAGGACATTGTCTTGATTAGTCATTGGTGTGGGAGAACCC~~
~~AGGTCACTGTGGGTGGTGCCATCCCTGGGCAGGTAGCCCTGTAGGTATATTAGAAAATAAGTAAATAAAA~~
~~CTAGGCGAAACAAGTCAGGGTGAGCAAGCCAGTAAAGTTCCCGCATGGTCTCTGCTTCAGTTCCTGCTCT~~
~~GAATTCCTGCCCTTCAAAATGGACTACAACTGTAACTGTAATAAGCCCTTTCCTCACCACAAGTAGCTTT~~
~~TGATCATGGTCATTATCACAGCAATGCAAACTTCCCAAGAGGAGAGTTCACATATGCTGACAGCTTACCA~~
~~GACCACCTTACCTTTGGATAGATTGGAGAAGAGACAATGTCAGGTCAGTGATGGGCGGGACCACATCTTG~~
~~CCCAGGGCTGCTCAGCTGTGTATGCCTCTTGTCCCCTATGTAAGTCTAAACCTCACCCAGGACCTCAAAG~~
~~ATGGAGCTAGGGAATCAATGGGCCTCTTTATTTGTTCCTCAGCTTGTAACTACTAAATGATCGTTAATTG~~
~~GTGTGCTGAGGGCAGGTGGCTCAGCCTGGCTTGTTAGGACTGCCAGAGCCCAGGCCTTGACACTAATTAC~~
~~TCCAGAAATACCTTCAAGTTAAATAAGCCGTTAGAATAACTCATAAAAGCACTTCAAGCACTGAGCTTAT~~
~~AAAGGGTCATGAAGGACAGAGATCAAGAGTAGCCAGATTGTCTTACTCGATGACCTATGGCCATAAAGAG~~
~~ACACCATGGCCATGACAACTCTTATAAGATTTTAATTTAATTTAATTGGGGCTTACATTTAATTGGCTTA~~
~~TATTTTCAGAGGTTTGGTCCATCATCATCAAGATGGAAGCCTGATGACACATAGGCAGATGTGGTGCTGG~~
~~AAAAGGAGCTGCAAGTTCTACATCAGAGCCCACAGGCAGCATGAAGAGAGGGTGCCACTGGGCCTGGCTT~~
~~GGGCTTTTGAAATCCCAAAGGCCACCCCTAGTAACACATTTCTTCCAACAATGAGGCTATACCTCCCAAT~~
~~CCCTAATGGCCACACATTTAAATCTTTGAGCCTAAGGGGACCACTCTTATTCAAAACCCCACACAGATGA~~
~~AGAGAAGTAAGGAGGGGCCAAGGGGTGGAAGATGCAGGAATGTCCCTATGGGACAGACACCCACCAGGCA~~
~~CACCAGAAATCTTCCTCATCATTCAAGTCCAGAGCTTTTAGGAAGTTTCATTACATAATCATGATCGATT~~
~~AAACTACTGACTGTCGGTGACTGAATTCAGCCACCATCCCCTCTCTCGTACCTGGAGGTCATGAGTTGAT~~
~~CTGGGAGGTCCAGCCCACACTAATCCTGTGCCTGGTCTTTGTGGTGACCAGCCCATCTGAACCTGCAGAA~~
~~GTTCCCATATGATCAGTCCCCATTTGAAGCTATGGAAACCACCCTTTCAAGTCTCTATGCTGGAAGATA~~
~~AACATCTTGTCACACAAAGAAGCACATGAAAAAAGCAAAAGGAAGTCAGCACGGGCAAGGCAATTTCTTT~~

~~TCTGCAAAAAGAAGCATGCATCAGAAGCCAAACCAGGCTAGAAAGCAGCACTCTTAGCCAAAATGGCCTC~~
~~CAGCTCTCATCTCTGATGTAAATGGTGACTGTGTTTCTTCCCGTTGGTGGAGCTCAACTTCATAACTGA~~
~~TGGGATTGACATTTGACGCAATTAGCTAATGGGTATAAAGAGGGAGGAGGGGACTTGGGAAATATGGGTC~~
~~CTTGTTAGGCTAGCTACCTGGGTAGAAAAACACAAAACAATATTTCTTACACCCATCTGAAGCTATGATT~~
~~TGGCTGCATCTCAAATGAACTGGATCAGACTAGGGTAGTGTTTTATAGCAATTTCCCCTGAGACAAAAAT~~
~~CCTCCATCTTGGAGAGAATCCTAGAAAGTCATGGATTTTCTAAAAAGGGGTGCCAGCTAAAACTCAAAA~~
~~GCTCCATCCTGCAGAGAAGCTAGTTAAAACTCAGGAAGTAAACAACACAAAACTTTCAGGAAGTTTCTGA~~
~~AACTGATCACATGTGCTAGGCTCCTCCTTCCTCAAATGTATATAAGCAGTAAAAACTGCTGAAAGACACT~~
~~CTTGGGCCAGTAAGCCATATGGAAAAAAACTCAGACCAGCAAGCTACTTGTAAAGGACCCTCCCCAGCCC~~
~~ATGGAGCTGCCTACCTGCTGCACAGTATGCTCCAGGTTCCCAGTTTTCATGAGCCGTTGCTCACACTAGG~~
~~GTGGGCTTCGGTAGTTAAGTCGTCTCGAGTCACTTCTGCTCCTGTAAGTAACCCTCTGTGGTGGTGTGAA~~
~~TGAGAACGGCCCCATAGGTTCCTGTGCTTGGATGCATGGTCATCAGCCGGTGGAACTCTTTGGGAAGGAC~~
~~TAGGGGCTGTGACCTTTTGGAGATGTGTCACCAAGGTTTCAAAAGTCCATGCTATTCCCAGTGTAGGTTC~~
~~TTTCTCTCTCTGCCTCCTACTGTGGATCAAGATGCAAACCTTCAGCTACTGCTTCAGTGCCAAGCCTGCT~~
~~TACTTCCATGCTCCTTGTCATGATGGTCATGGACTTTAACCCTCTGAAACAGTAAACTCTCAATAAACTC~~
~~TTTCTGTATGTTGTCTTGATCATAGTGGCTTATCACCGCAATAGGAAAGTAACTAAGACACCTTCACTCA~~
~~TACTGCTATGAGTAACCCAAAGAACACTCATTGTTTCATCAAGCTGGACTTTGGCAGTCTCTTACTGTGA~~
~~TCCATCATTGGTTCCCTATCTGGGCTGAGTAGATATTTGTTCATGTCTCTCCTTTCTTGCCTCCTACCAG~~
~~TCATAATAAAGGAAGGTCACATGGCTTTGCCATCTTAAGGCCAAATACTGGAAGATTAATTTTAATT~~
~~GTTAATTTGACTGAATTTAGAATCAGCATGGAGGAGCTGGAGAGATGGCTCAGTGGTTAAGAGCACTGGT~~
~~TGTTCTTCCAGGAGTCTGAGTTCAATTCCCAGCAACCACATGGTGGCTCACAACCATCTATAATGGAATC~~
~~TGATGCCCTCTTCTGATGTGTCTGAAGACCGTGACAGTATACTCACATATATAAAATAAATAAATCATTA~~
~~GAAAAAAAAAGATACCAGGCAGTGGTGGCCTTTAATCCCAGCACTTGGGAGGCAAAGGTGTGCTGATTAA~~
~~GATGTTTCTAGACTGCTTGCTGACAGGAGCGTGATAGAGCTGTCTCATGAGAGGCTCTGCCAGTGCCTGA~~
~~CAAATACACAAGTGGATGCTCACAGCCTTCCATTGGACTGAGCACAGGGTCCCCAATGAAGGAGCTAGAG~~
~~AAAGGATCCAAGGAGCTGAAGGGGTTTGCAGCCCCATAGGAGGAACAATATGAACCAACCAGTACCCCCA~~
~~GAGCTCCCTGGGACTAAACCACCAACCAAAGAGTACACATGGTGGGACTCATGGCTCCAGCTGCATATGT~~
~~GTCAGAGGATGGCCTAGTCAGTCATCAATGGAGGAGAGGCCCCTTAGTCCTGTGAGGACTCTATGCCCCA~~
~~GTATAGGGAAATGCCAGGGCCAGGAAGTGGGTATGGGTAGGTTTGTGAGCAGGGGGAAGGGGAATGGGAT~~
~~AGGGGGTTTGGGGAGGCAAAATCAGGAAAGGGGATATTAACATTTGAAATGTAAATAAAGGAAACATCCA~~
~~ATAAAAAATAATTGTTCTGGGCTGGTGAGATGGCTCAGCAGTTAAGAGAACTGACTGCTCTTCCAGAGGT~~
~~CCTGAGTTCAATTCCCAGCAACCACATGGTGGCTTACAACCATCTGTAATGAGATCTGATGCCCTCTTCT~~
~~GTTTGAAGACAGCTACAGTGTACTCACATACATAAAATAAGTAAATAATTTTTAAAAGGAAGGAAGGAAA~~
~~GAAAGAAGGAAGAGAAATAATTTCACTTCCATTTGGGACTCAGAAAAAGTATAGATATCTGTCAAATAGA~~
~~AAAACACAAATTTGGTAAAAGAACAAAAAAAAAATTGTTCTTCTCATACACTGAAAAAAAAAAAGATGTT~~
~~TCTAGATGACTTTAACAAGGAGAAAAGACCCATGTGAATGGGAGGGAAGGGGTGGAGAACACCATTCTGG~~
~~GGACTGGAATACCAAAATAAATAAAGAGAAGAAAGTCAAAGCCAGGCATGGTGGCACACATATTATTATT~~
~~ATTATTATTATTATTATTAATTTAAAGTATGACTGCTCTGATGCATATACACCCTCAGGCCAGAAGAGGG~~
~~CACCAGTTCCCTTATAGATGGTTGTGAGCCACCATGTAGTTGCAGGAATTGAACTTAGGACCTGTGGAAG~~
~~AGCAGCCAGTTCTCTTAACCTCTGAGCCATCTCACACACATCTTTAATTACATCATGCAAAAGGCAGAGG~~
~~CAGGAGGATCTCTGAGTCTGAGGCCAGCCAGTGAGCTCCAGGGCAGCCAGGACTGCTCTAAAGACTGTCT~~
~~CTAACCCTCTCCCCCCACCCTGCAAAAAAAAAAATAAAAAATATAGCTGACCATCAACATTCATTTCTCTC~~
~~TGCTTCCTGACTATGCCTTTTGGGTCTGAGGTGAGGAGGTGCAGTAGCTCTGCTGGCTACTGTGTGCTCA~~
~~GGTGGCTCAGCAGGTCACCTGTGCATGGAGCTCAAGGCCAGTTCAGTTTCAGCATCCACACCATTCACGT~~
~~TAGGACCAGTGGTCCTTCTTCCATTCCCTCAAGTCACTGCAGGACTAGGCACCTCCTCCCCACTGAGGCC~~
~~AGATGAGGCAACCCAGTTAGGGGAACAATATCCACAGGCAGGCAACAGAGTCAGGGTAAGCCTTAAGAAG~~
~~CATTCTTGGGGCTGGGAGATGGCTCAGTGGTTAAGAGCACCGACTGCTCTTCCAGAGGTCCTGAGTTCA~~
~~ATTCCCAGCAACCACATGGTGGCTCTCAACCATCTGTAATGGAGTTTGATGCCCTCTTCTGGTGTGTCTG~~
~~AAGACAACTACAGTGTACTCATATACATTAAATAAATAAATCTTAAAAAAAAAAAGCATTCTTTACAGCA~~
~~CGTTGGTCAATACAATGCCCATGTCCAGATTACAACCTGATCCTGGTCACAGAACTCAGCAGCATCTAAG~~
~~CATATCTTGCACAAGACCAACCCTAGCAATGGCCTCTGCTCCAGTTTCTGCCTTGAGCTCCTGTCCTTTC~~
~~TTTGATGATGATGTGTGATGTGAGTCAAGTAAACCACAAGTTGCTTTGGCTATGGTTTTTATCACAGTGA~~

~~TAGCAACCTAACAAAGACATTGGCCAAAGTCCATCTTAAATCCCCTAACACTTTGGGGTTCCTTGCTCCT~~
~~TTCCTAAAGCTCTACTCCCCAGCACAAGGTTGCCTGACAAACATGGTGGCTGACCCTATCTGAGCTGCAT~~
~~GGCTCTAGCTCCCGGCCACATGGATGCCTGCCACCCGGTGGCTAACCTGTGAACTGCCCAGCTCAAGCTG~~
~~CAGGGCTATTCTCCAGTCCCCAGGCAGCTTTGAGGTTTATGGCTGACCGGCCCCGGGGTTTTCTTTTTGA~~
~~ACTCTAACTGTCCTAGGCTTCCATTTGTGTGTATGTCCTAAATTATTTTATTAATAACCCTAAGGAAGAA~~
~~CCCTGATTCCCTAGGCGCCATAGAATGGCTCTCCTGGGGTTCCCCAAGATCCCCAATAACACATCTTTCC~~
~~TACTTAATTATTTAATTGGCAGAGAAAATGAACCCATTCCCATACTTCTAGCTGCCATAGTCCTGGGGCT~~
~~ACAAGGCCTCTGGCACCTGGTCTAGCCTGGGGTTGGTTCCAGGAACTGTGGCGAGGTATAGGCATGAGAT~~
~~GAGGGTGGCTCACAGCTTTAAGGAGCCTTGAGTGGAGGTGCTGTCTTCAGAGGCTCCAGCCTTTCACTGT~~
~~TACAGAATATCTGATCACACTGTGAACCCTAAGACTGTGTTACTTACTGGAAAACCTGTTTGTAGTTGTG~~
~~GCAGCTCTTAGCACACAGCTCTAATCCCAAACAATGACAGTAAAGTAAGTTTGTAGAAGGAAGCACCCAT~~
~~GTTTGAAAGTGATGTCTACCTAATTGAGTGGCAGGTGAAGTGAAGAATCAGAGAAGGATCTGACAGAATA~~
~~TGATCTGCCCAACTCTCACAAGAGGAAAGGGAAGCTACTTGAGGGGCAGCGCAGAGAAAAGGAAGGAAGC~~
~~GGTTTTACTGCGACAGTTGTACAGAGGCAGATTGCCGAGTGAATAAATTAGACACAAGTGAAGACAGGAG~~
~~CCAGAGAATGAGGACCCAGAAGATTACAACAGATTGCCAAAGTTAGTAGGAGTTCAAGCAAAACAGTCAA~~
~~GAGGCCAAGAAAGAAGCCAGCTTGAATCAGTCAGTGTGGAGAGTGTGGAGAGTGTGGAGAGGAGTTTTGA~~
~~GCCAGAACAGCTGAGTTGACCAGCTCAAAAGAAGCTAGAAAGGGTGAGCTTATTCAGCAACAAGTCTCAG~~
~~AGGCTGAAAACATTCTAGGCCTAGATAAGATTCTACAGAGGCTAGAAGCTTCCAGGACTAGGCCTATTTA~~
~~GCAAACAGAGGCAGTAGGCCTCCAAGACAACAGTCCCAGCACTAAGAATTTTATACATTCTCAGCTGGCT~~
~~GTGGTGGTATGTCCACCACTGCAACCCTTCATTTCCTTTCTGGTCCATTTTATCTTTGTTTTGTTTGTTT~~
~~GTTTTTCCTTTTTGTCATTTTTGCTTTTGGTTTCCTACTAAGAAACTACTGTTTAATCCAAGATAGTAAA~~
~~AATGTGTTTCCCCTTAGGAGTTTTATATCTATATTATCTCTGACCTTTTTGAGCTAAATTTTGAATTCAG~~
~~CATGAGACTCTTAAACAAGAACCACCAAGGTAAAGCTAGTGTGGCAATATAATGCTAGCTACTGTAAGGC~~
~~TAAAGCAAGAGAAGCTTCCCTGGATAATTCAGCAATCTTTTATTAACAAGAACCAGCGTTTACGCTTGAT~~
~~TTTAGCCACAAAGGAGAGAAGTAATACACTCTTACAAACATAAAGATTTTTCAAGGAAAATTTTAAGTTA~~
~~AATTTTCCTTTCTACAAAATTAGGGCCAGCAGGATAACTTAGTGGGGACCTGGGGGTGGGGGTGGGGCAT~~
~~GTCTTTTGTCCTGCACTCCAGAGGCAGAAGGAGGGGGATCTCTGTGAGTTCAAGACCAGCCTGGTCTACA~~
~~CAGCGAGTTCCAAAACAGCCAAGGCTACAAGGAGAAACGCTGTCTAGGAAAACAGACAGTTAGTTTAGGG~~
~~GCCACTTGACTGTGAGTTCAATTCCCAGATCCCACATGATTGGAAGAAACAAATTTCCCCAAGTTTTCCT~~
~~GTGATCTCTTCATGCACACATACACCTTTGTCCCAGAGATGCCCGTCTGTGGTTTCCTAGCACTGGATTA~~
~~CTGGGAAAACTCACCTCCCAACCATCCTACCCAGCATTTTGTACTATCTCCTCAGTCCTGCCGTGCGCTA~~
~~AGGCAGGGCCTTCTCCAGGTCGTCCCGGCTGTCCTAAACTTGGTATGTAGACCAAGCTAGCCACGACCTC~~
~~AAAGATCCTTTTTCCTGTCTCCTGGCTGGGGTTTTAAGGTGTGTGCCACCACGCCCGGCTATTCTGCTTC~~
~~TTGATGCAGGGCCTCCACAGTAGT~~ACAGGCTAGCCCTCGGCCTCCTGCCTCCATTACTGAGATTACAGCA
ATTTATCGGTGTGCCGTTGTATACGGTATGTCCTTTACTTGTTTAATCTAGACAAACTGCTTGCATTTCC
GAAAAGACCTTCAGCTGGAGCTACAACTCGCTTCTCTGTAATTACCCGACACCTGGCAATGCCTTCCCGC
CCCGAACCCCAAAGTCAACACGGCGGTCCCAACTCACGCCTCTCCTACAGGACCTGTGAAAGGCCCAAGG
GAGTGGGGCAGAACGGGCTGACATCAGAGTCGCTACCCAAACTCCGCACAAGTAGGAGACTCTGGCCCGA
CGTGCCCTGCCGTGTAGAATTCACCCTCTCCTTCCCGTCTACGTTTCCCACGACCAGGATCTCACTACAC
TTCCCAGTACCGGAAGCCTGAGCGCAACAAAGATGGCGACTGCGGAAGGGGCGGAGGGCGTGGCCTCCGT
CCGGGAGCACTGGGCCACCTAGCTTCCGGCGGCGCGCCAGTGAC**GTC**AGTGAGCGCGCCAGGCCGCGTGG
AGGCCGTAGGCTGATGGAGGTCAGTGAGGGTGATCGCCGCCGCAGTCTGGCGTCCTGGAGAGACCGGGTT
TCTGAACGTGGGTATCCGAGACGGGAAGCGTGGCTGGGAAGGGCAGCAGTTAGTAAGGACCGGCGGGGCT
GTTGTGGGGGGAGCCAGGAAAGGGACTCTCGACACCGTGCCCCAAGGGCGCTGTTCGGTTGCAAATTCGG
TTCC

Stop codon of Tbrg4 gene

Tbrg4-3’UTR

Inserted Wap-3’UTR

Tb-Wap-3'UTR (Δ1,080, 130bp insertion)

CCACTGGTCGTGTAAAGGCAGGAAAAGACTCCGTAGTCCATGTGACCTGTCTCTACTACCTTCTTCCAGG
TCCCATATTATGAATGGCTGGATCTGAAGTCTGAATGGCAGAAAAGCGCCTACCTCAAGGACAAGATGCG
AAAAGCAATGGCTGAGGAGCTAGCCAAATGACCT**GCCGGCCCTGGCATCCCTGCCTGTCGGGAGTGACCA**
GCCCAAGTCTATACAGCAAGACCCTTCACTCCTGGATCCAGAGAGAACATAATGCTTTCAATCTGCTGCT
AATAAAAATCCATTTGGCTTTATG~~CCTTACCCATCAGTTCAGACTGCTCCCAAAGACCTGACTGCTCAGG~~
~~GACCCAGCAGTGAGTGTTGGGCAGGCCAGAGTTGTCATCCAACATCAGAGAGATTAACTGTGTCTGTAGG~~
~~ACGTGGTCAGAGTAGGGGGTGCTCAGCATCTCCAAGGAACGGGTGGTCCTAATAAATCCTTTGGTTTGGT~~
~~GGAAGCCAGCTATCTTCCCAGGCTCTGTATCTATCTCCTCTCTCTCGTATTTTGCACACACATACATATC~~
~~CTCTTCCCTAGCTCTCCAGGATTCTACCGAGTGGGCTCTCTTTCCCCAGCTCCCCCATAATGCCTGTGTG~~
~~CTGTGGCACAGCAGAGCAGGGCTGACTTGAGAGACAGAAAACTGAGACTTAAATGGGTAGGGTCCTGGAC~~
~~ACTTGAGCTTCCTGGGAAATGTACTTGGAGTGTATAGCAGAGTTGTGATTACTATCTTGAGAGGTGCCCA~~
~~CACCACCCCCCACCCCCACACCCCCGCACCCCCGTGCCACTAGAGAACATCTGAAGGAACAGGTGCTGT~~
~~GAAGACATTTGGAAGGCCCTAGTTCTGGGACAAGAGTAAAGTCCACTTGGCTGATGCTACAAGATCCATG~~
~~GCCAGACTCATTCTCATGACTACTACAGCCAACCCAGGACCGCCCTTCAGGTGGAGCCCTGGGTCCCCT~~
~~GCAGCTCTGAGTAGCAGCAGGATGTCTACCTTCTACCCTCTGCAGGAGTCTCCCATAGATAAGGCTCGCC~~
~~CAAAGGGGAGACTTGCAACAAAGGGGGTTATTACCCTACTTGGTACATTCCTCAAAGCACCAGTCCTCTG~~
~~TGTGACTTAGCACTTCTATTGTCCTGACAGACTCTTGGTTACCACAGAGGTGAGGGAGAAGCAGAAAAGC~~
~~GGCTGGAACTGGCTTACAGGAAGGAGCAGCTGGCCACAACTTGTCTTAAGTTGAGGTGGGTTGGTAGTGC~~
~~TGACAACCCAGTTGGGGAAGTCTTGTGTATGAGGAAATAAAAGTTTGTGCTGCTCACCACAT~~GGGCCACT
GGCTTTTCTTTTGAGCTAATGGAGATCTCCAGCTAATACTCGGTGGCGTGGTCAGTGGTGGAACTACTGC
AGAGCCACCC

TSS of *Tbrg4* gene

CREB

ΔCRE (Δ22)

CACAAGTAGGAGACTCTGGCCCGACGTGCCCTGCCGTGTAGAATTCACCCTCTCCTTCCCGTCTACGTTT
CCCACGACCAGGATCTCACTACACTTCCCAGTACCGGAAGCCTGAGCGCAACAAAGATGGCGACTGCGGA
AGGGGCGGAGGGCGTGGCCTCCGTCCGGGAGCACTGGGCCACCTAGCT~~TCCGGCGGCGCGCCAGTGAC~~**~~GT~~**
CAGTGAGCGCGCCAGGCCGCGTGGAGGCCGTAGGCTGATGGAGGTCAGTGAGGGTGATCGCCGCCGCAGT
CTGGCGTCCTGGAGAGACCGGGTTTCTGAACGTGGGTATCCGAGACGGGAAGCGTGGCTGGGAAGGGCAG
CAGTTAGTAAGGACCGGCGGGGCTGTTGTGGGGGGAGCCAGGAAAGGGACTCTCGACACCGTGCCCCAAG
GGCGCTGTTCGGTTGCAAATTCGGTTCC

Supplementary Table 5. RNA-seq data of WT and Wap-Tbrg4 mice (separate Excel file).

List of all genes with FPKM value for exon and intron in each replicate at L10 mammary tissue from WT and mutant mice.
